# Design and application of a modular and scalable electrochemical flow microreactor

**DOI:** 10.1007/s41981-018-0024-3

**Published:** 2018-11-22

**Authors:** Gabriele Laudadio, Wouter de Smet, Lisa Struik, Yiran Cao, Timothy Noël

**Affiliations:** Department of Chemical Engineering and Chemistry, Micro Flow Chemistry & Synthetic Methodology, Eindhoven University of Technology, De Rondom 70 (Helix, STO 1.37), 5612 AP Eindhoven, The Netherlands

**Keywords:** Electrochemistry, Flow chemistry, Scalability, Reactor

## Abstract

Electrochemistry constitutes a mild, green and versatile activation method of organic molecules. Despite these innate advantages, its widespread use in organic chemistry has been hampered due to technical limitations, such as mass and heat transfer limitations which restraints the scalability of electrochemical methods. Herein, we describe an undivided-cell electrochemical flow reactor with a flexible reactor volume. This enables its use in two different modes, which are highly relevant for flow chemistry applications, including a serial (volume ranging from 88 μL/channel up to 704 μL) or a parallel mode (numbering-up). The electrochemical flow reactor was subsequently assessed in two synthetic transformations, which confirms its versatility and scale-up potential.

**Electronic supplementary material:**

The online version of this article (10.1007/s41981-018-0024-3) contains supplementary material, which is available to authorized users.

## Introduction

In the past few years, electrochemical transformations have received renewed interest from the synthetic community as a powerful activation mode to enable versatile organic transformations [1–31]. The application of electrons as traceless reagents avoids the use of hazardous or toxic oxidants, providing milder and more sustainable processes [7, 8, 12, 19, 22]. In addition, key electrochemical parameters, such as electric current and potential, can be easily tuned, providing an improved functional group tolerance and selectivity compared to classical thermal approaches [1, 3, 7, 12]. Even though the advantages of electrochemistry appear numerous and many remarkable procedures have been developed employing this technique, many synthetic organic chemists have been discouraged to apply this technique. This can be attributed to the need for specialized equipment and to the knowledge gap of most researchers in this rather esoteric discipline [2, 9]. In addition, electrochemical setups are often affected by process-related problems, like mass- and heat-transfer limitations, and by electrodeposition of organic substances on the electrode surface [32–40]. These drawbacks limit the reproducibility of electrochemistry and can hamper dramatically both its widespread use and its scalability beyond a laboratory scale [2, 7, 9, 19].

From its advent in 2012, our laboratory has always been interested in the development and manufacturing of novel flow reactor technology to overcome technological limitations in organic synthetic chemistry, such as photochemistry [41–44] and gas-liquid transformations [45–48]. We felt consistently that a “Do-It-Yourself” (DIY) approach was beneficial as it leveraged a fundamental understanding of the technology [49]. This further enabled us (i) to reduce the overall capital investment, (ii) to repair setups quickly, (iii) to customize the design to our specific needs and (iv) to exploit the technology at its full potential.

We anticipated that also electrochemistry required a technological impetus to overcome the hurdles as described above. Indeed, most of the limitations associated with organic electrochemistry can be overcome by performing electrochemical reactions in continuous-flow microreactors. Specifically, the confined dimensions of micro-flow reactors (up to 1 mm interelectrode gap) allows to reduce the Ohmic drop, to minimize the total amount of supporting electrolytes, and to increase mass transfer from the bulk solution to the electrode surface [32–40, 50–54]. In addition, due to the continuous nature of these reactors, generation of local hotspots can be prevented. For these reasons, several electrochemical continuous-flow reactors were developed, commercialized and successfully deployed in a wide variety of electrochemical reactions [32, 34–39, 51, 55–62]. However, despite these great advances, we felt that a cheap, scalable and modular electrochemical flow reactor was still missing. In this article, we disclose our efforts towards this specific goal and we benchmarked the electrochemical reactor in two relevant electrochemical transformations.

## Results and discussion

### Reactor design

At the outset of our design efforts, we defined the following design criteria for our electrochemical flow reactor:

i) flexible reactor volume which allows to carry out the reaction both at small and large scale;
ii) variable spacing between the electrodes, which can be readily accessed through adjustment of the gasket thickness;
iii) simple and flat electrode design to avoid complex machining requirements;
iv) high modularity in combination with easy exchangeable components;
v) inexpensive and solvent-resistant reactor materials;
vi) safe operation of the reactor where the wet part and the electric parts are adequately separated.

**Fig. 1:**
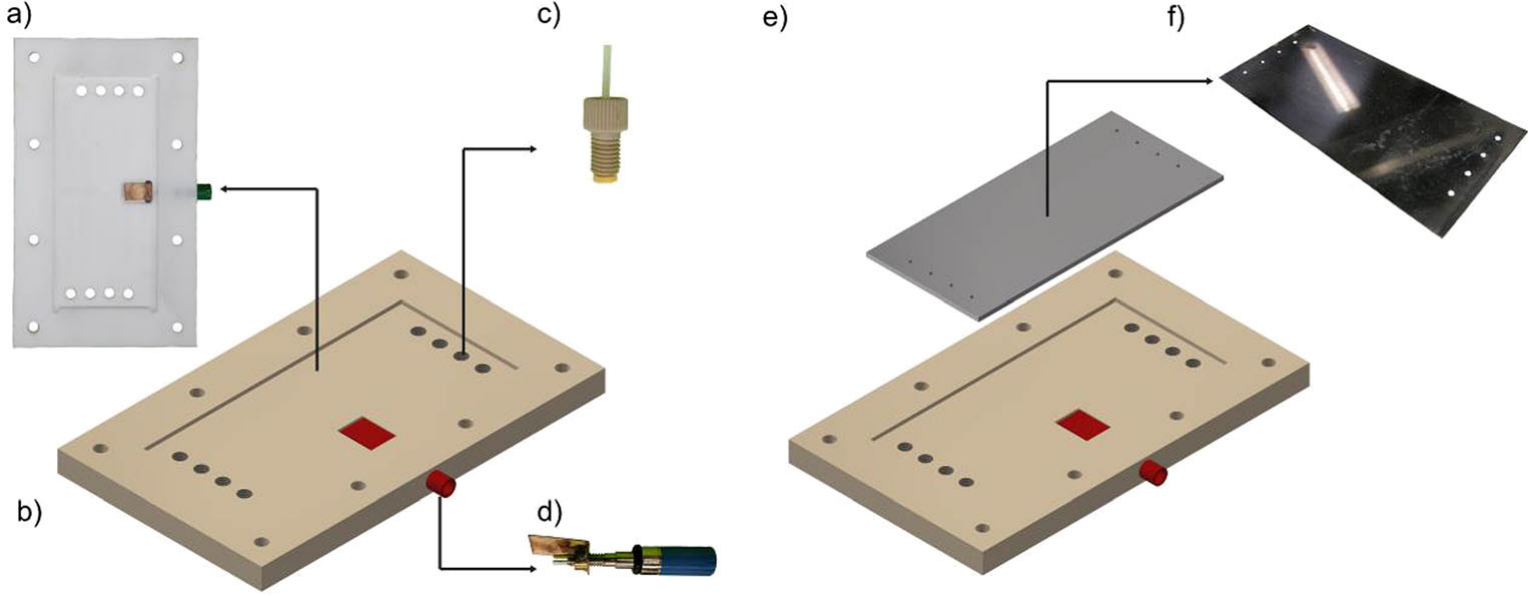
Individual parts of the electrochemical flow reactors: **a** PTFE casing; **b** electrode casing; **c** Super Flangeless Fitting; **d** electric contact; **e** the electrode plate; **f** electrode

**Fig. 2:**
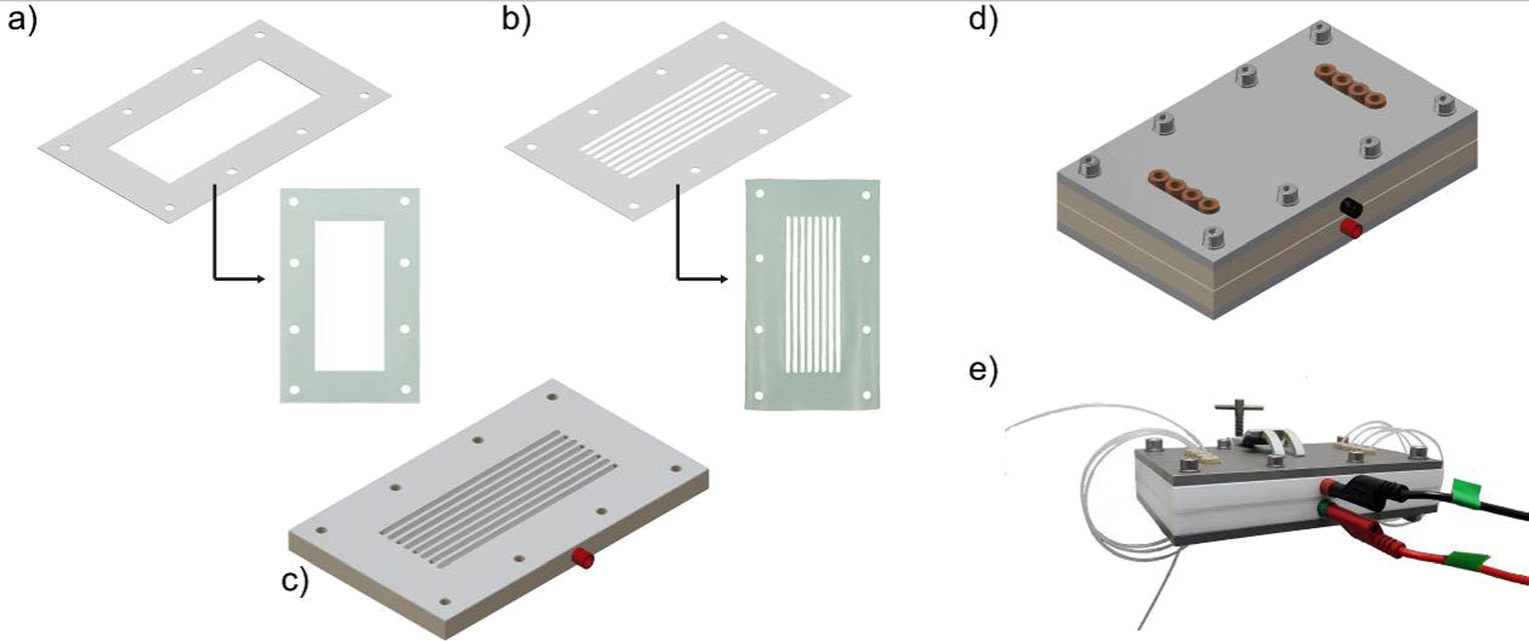
Schematic representation of electrochemical flow reactor. **a** open-channel gasket design; **b** 8-channel gasket design; **c** bottom plate with electrode and 8-channel gasket; **d** schematic representation of the complete device; **e** picture of the assembled electrochemical flow reactor with banana cables to establish the electric connections

For the electrode casing, an easy-to-machine rectangular insulator (PTFE, polytetrafluoroethylene; 160 mm × 95 mm × 10 mm) was chosen, which is solvent resistant and can be compressed between two stainless steel chucks using 8 screws (four 6 M × 400 mm screws + four 4 M × 400 mm screws) (Figs. 1 and 2). To introduce the liquids into the reactor, we used Super Flangeless Nuts (PEEK, 1/4–28 Flat bottom, for 1/16”OD) which enable a distributed injection of the reaction mixture over the electrodes (Fig. 1). Through this design, the contact between the reaction mixture and the insulated electrode holder is minimized. In addition, it also circumvents the need to include in the design a complex and difficult-to-machine flow distributor [63, 64]. The connection between the electrode and the power supply was achieved via a threaded connection positioned at the insulating plate. Constant contact between the electrical connection and the electrodes was ensured via a spring. From our experience, we found that this strategy represents an excellent alternative to the classical soldered electrical contacts.

Flat rectangular-shaped plates (120 mm × 55 mm × 2 mm) were used as electrode material and could be readily fit into the PTFE casing. In order to avoid the use of complex and expensive electrodes (e.g. machined channels in the electrode plate), only small holes were drilled to establish the microfluidic connections. Between the electrodes, a PTFE gasket was placed which can be adjusted in thickness (*d*_*G*_ = 0.25–0.5 mm are used in this manuscript) and shape, e.g. an open-channel gasket (110 mm length × 45 mm length) or an 8-channel gasket (106 mm length × 3 mm width per channel) (Fig. 2a–b).

### Reactor characterization

**Fig. 3:**
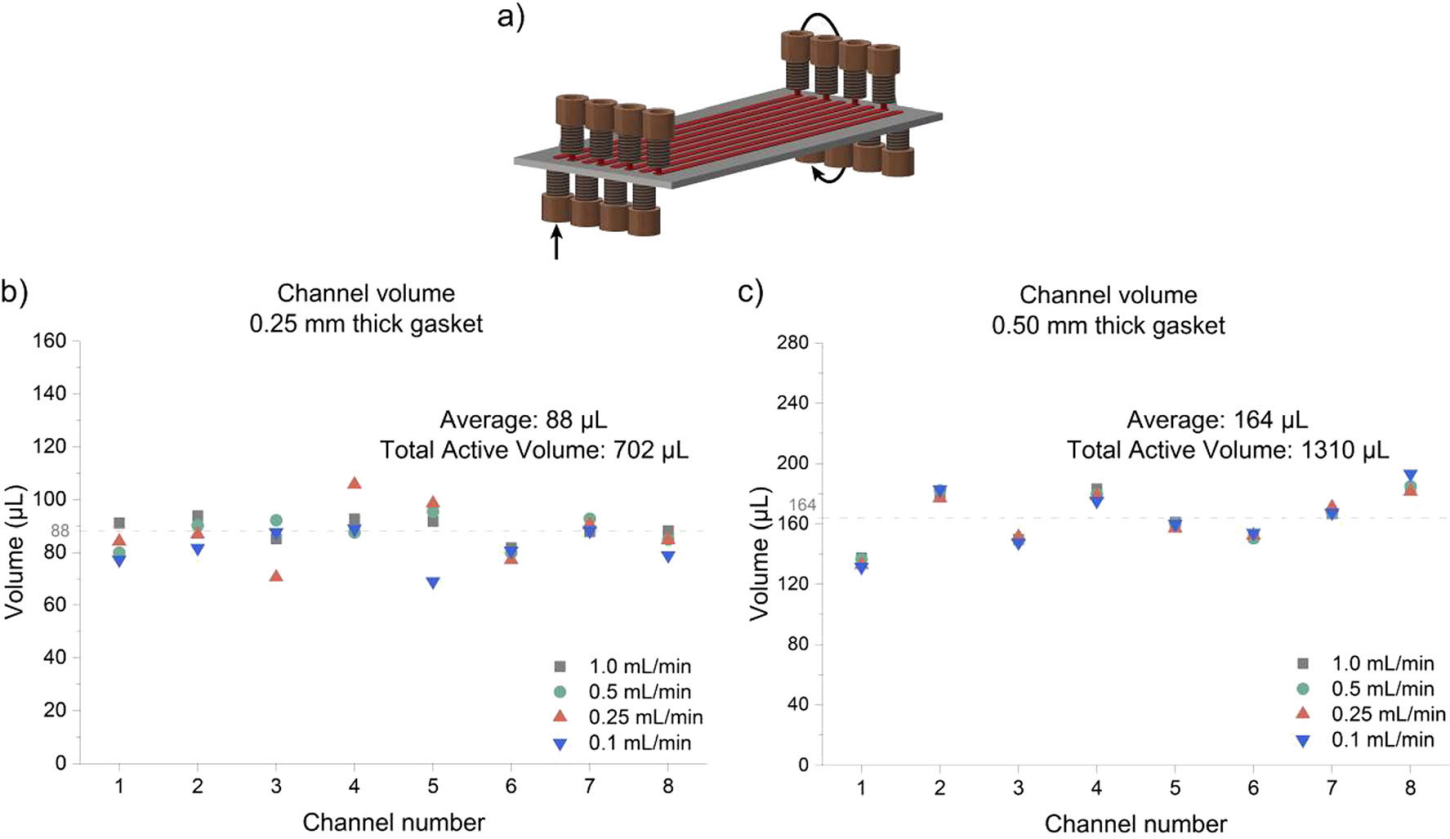
Reactor volume measurements at different flow rates. **a** Schematic representation of the individual channels (in red in the figure); Obtained results for the (**b**) 0.25 mm gasket; and the (**c**) 0.50 mm gasket

During the course of our investigations, the 8-channel gasket was preferred as it enables a better fluid distribution over the electrodes and a more narrow residence time distribution. In contrast, the open-channel configuration displayed bad mixing behavior and was not further pursued. Notably, using the 8-channel gasket (*d*_*G*_ = 0.25 mm) equipped with stainless steel electrodes (SS), the reactor can be rapidly reconfigured giving access to a flexible reactor volume ranging from 88 μL/channel up to 704 μL when all channels are used in series (Fig. 3). Furthermore, a result within the 88 μL reactor can be readily scaled by a factor of eight through use of all the channels in parallel (numbering-up). This flexibility in configuration is a unique feature of our reactor design providing rapid access to a wide variety of residence times and reaction scales in a single design.

To elucidate the average residence time in the individual reactor channels, flow rates ranging from 0.1 mL/min to 1.0 mL/min were evaluated. As shown in Fig. 3, the volume of the individual channels averaged around 88 μL and 164 μL for the 0.25 mm and 0.5 mm thick gasket, respectively. The small differences between the channels can be attributed to the positioning of the flexible PTFE gasket upon closing the reactor. The standard deviation measured for both gaskets was below 10% (6.9% and 9.0% for the 0.25 mm and 0.5 mm thick gasket, respectively), which was considered acceptable (see Table S1 in the Supporting Information).

### Reactor performance

Next, we assessed the utility of this novel electrochemical flow reactor by examining its performance in two electrochemical transformations.

**Fig. 4:**
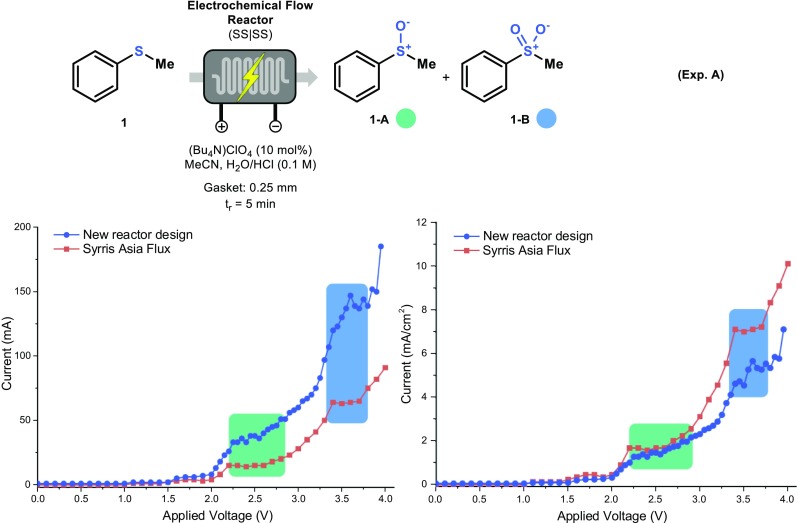
Voltammogram comparison for the electrochemical oxidation of thioanisole (**1**) to the corresponding sulfoxide (**1-A**) or sulfone (**1-B**)**, a** with the newly developed electrochemical microreactor and the commercially available Syrris Asia Flux. The two voltammograms represent the same experiment, one with the measured current (**b**) and the other one with current per surface area

As a calibration point, we compared the performance of our novel reactor design with a commercial electrochemical flow reactor (i.e. Syrris Asia Flux) in the electrochemical oxidation of sulfides, a transformation previously reported by our laboratory [65]. This reaction is particularly interesting as the selectivity towards sulfoxide or sulfone is governed by the applied potential, while hydrogen reduction is observed as cathodic reaction. We recorded voltammograms for this transformation in the two reactors as shown in Fig. 4. The voltammograms show two similar plateaus, indicating the oxidation towards sulfoxide (**1-A**) and sulfone (**1-B**) located respectively between 2.2–2.6 V and 3.3–3.5 V [65].

In addition, during this experiment, the temperature of the reaction mixture was constantly monitored via a thermocouple at the outlet of the reactor [66]. The temperature remained constant during the entire experiment, which proves that our microreactor dissipates efficiently the generated heat to the environment.

Next, we carried out a systematic evaluation of different process parameters, i.e. residence time, gasket thickness and electrolyte concentration. The different reaction conditions are listed in Table 1.

**Table 1:**
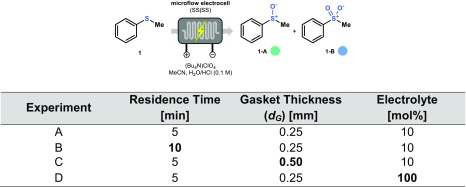
Screening of different parameters (B: Residence Time, C: Gasket Thickness, D: Electrolyte)

Reagents and conditions: Thioanisole (2 mmol, 0.1 M), Bu_4_NClO_4_ (10 mol% or 100 mol%), MeCN/HCl (20 mL, 3**:** 1 *v*/v, with 0.1 M HCl in H_2_O), Stainless Steel as anode/cathode, residence time: 5 min (at a flowrate of 0.15 mL min^−1^) or 10 min (at a flowrate of 0.075 mL min^−1^)

**Fig. 5:**
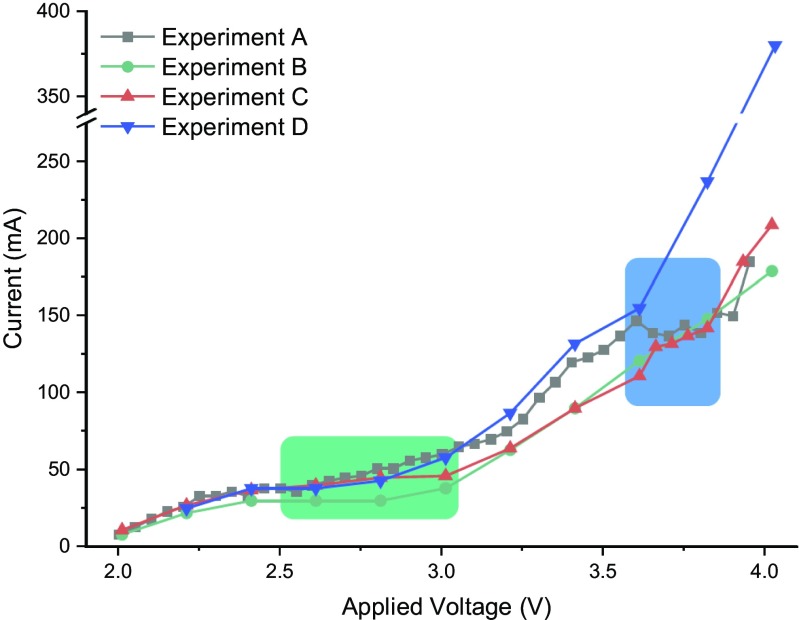
Comparison of the different voltammograms for the electrochemical oxidation of thioanisole (**1**). For the different conditions, see Table 1

For each of these conditions, we recorded a voltammogram which is shown in Fig. 5. The same trend was observed in all cases, particularly at low voltages. The first plateau is visible in all the different experiments (green box, Fig. 5), while the second plateau (blue box, Fig. 5) is not visible at a higher electrolyte concentration (Experiment D). This effect is probably caused by a faster degradation of the electrode at higher voltage in the presence of a more conductive solution, corresponding to the higher increment of the current.

**Fig. 6:**
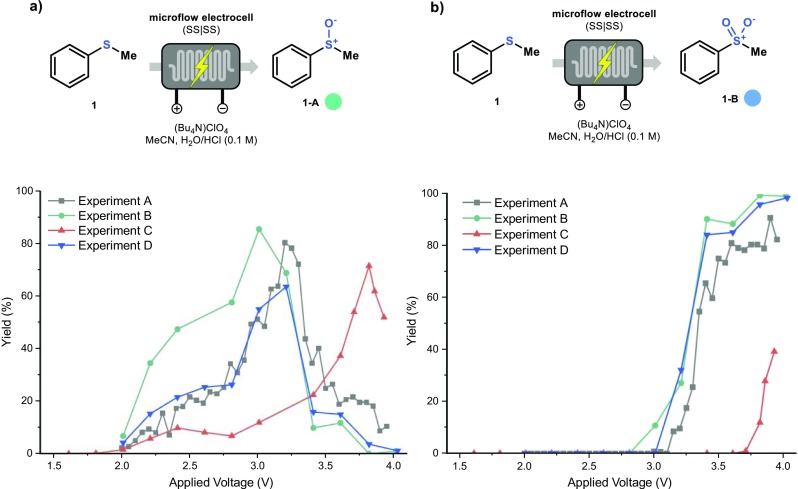
Comparison of the yield towards sulfoxide (**1-A**, **a** and sulfone (**1-B**, **b** for the electrochemical oxidation of thioanisole (**1**). For the different conditions, see Table 1. Yield determined by GC-MS with biphenyl as internal standard

Next, the conversion of thioanisole (**1**) to sulfoxide (**1-A**) and sulfone (**1-B**) at different cell voltages was investigated (Fig. 6a and b respectively). Experiments A, B and D follow the same trend, with a maximum conversion between 2.8 V and 3.2 V for sulfoxide and at 3.6 V for sulfone (See Supporting Information for more details). It should be noted that increasing amounts of electrolyte decreases the sulfoxide yield (63%, Experiment D). Notably, increasing the residence time (Experiment B) results in a higher conversion to sulfoxide (**1-A**) at lower voltages. Furthermore, a thicker gasket clearly shifted the respective transformations to higher voltages, even if the voltammogram results were similar to the other experiments. This observation implies that indeed inter-electrode distance plays a key role in electrochemical transformations: not only does an increased inter-electrode distance result in a higher Ohmic resistance but it also exacerbates the mass transfer limitations.

**Fig. 7:**
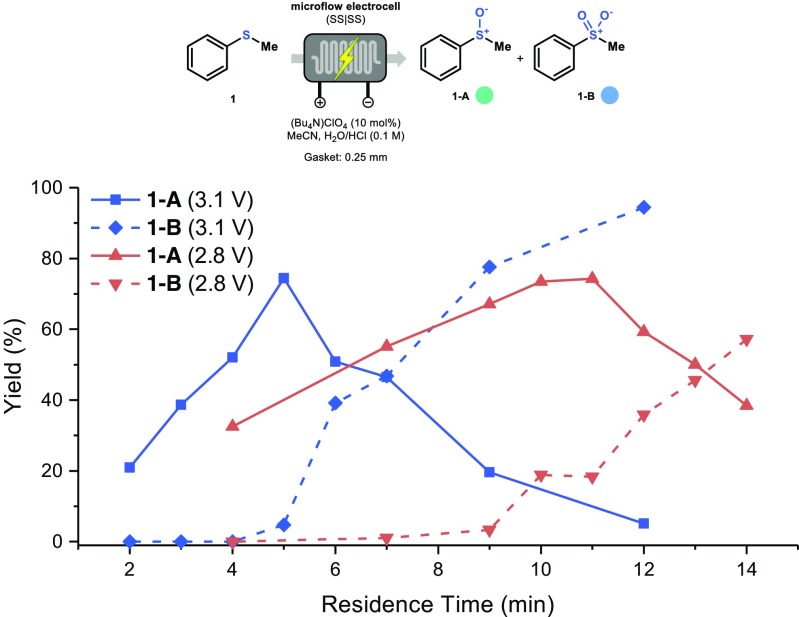
The relation between residence time and applied voltage and its impact on the electrochemical oxidation of thioanisole. Yield determined by GC-MS with biphenyl as internal standard

Next, we decided to further explore the inter-dependency of residence time and applied voltage in Experiment B. Therefore, different residence times were evaluated at 2.8 V and 3.1 V respectively (Fig. 7). At 3.1 V, the production of **1-A** increases until about 5 min residence time. At higher residence times, the yield of **1-A** drops and product **1-B** is formed instead. Interestingly, at 2.8 V a similar trend is observed, with a shifted maximum yield for **1-A** at 10 min, while prolonged reaction times affected negatively the selectivity. This result reveals a synergistic effect between reaction time and applied voltage.

**Fig. 8:**
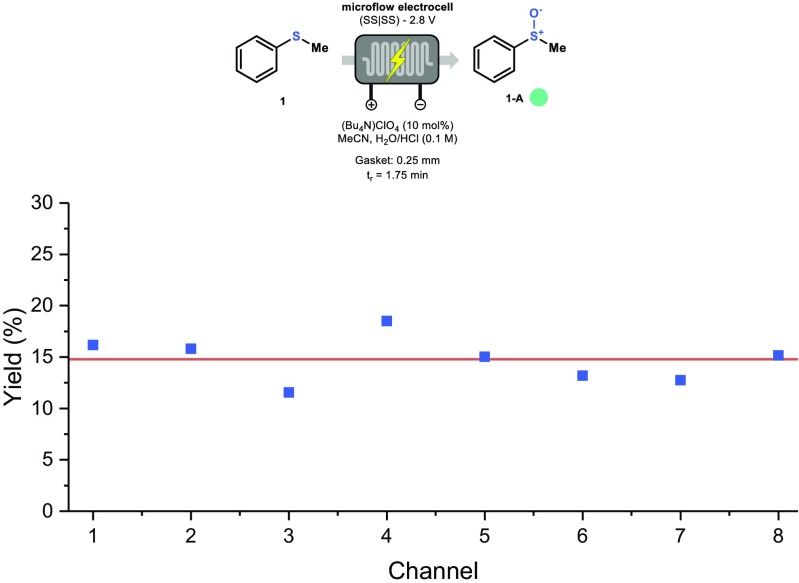
Relative deviations of the yield among the different channels in the electrochemical flow reactor. Yield determined by GC-MS with biphenyl as internal standard

Next, we set out to analyze the yield differences in the individual channels. A consistent performance is required in each channel if we want to use our reactor as a numbered-up device. Hereto, every channel was fed with the reaction mixture separately. We selected a short residence time of 1.75 min to maximize potential yield variations between the channels (Fig. 8). To our delight, a consistent performance was observed in all the channels with an average conversion around 14.7% (line orange in Fig. 8). Small yield differences can be attributed to the slight variations in channel volumes as discussed above (see Fig. 3b). Notably, this results also demonstrates convincingly that the entire electrode surface is equally active and that the stainless steel electrodes are homogeneously polarized.

**Fig. 9:**
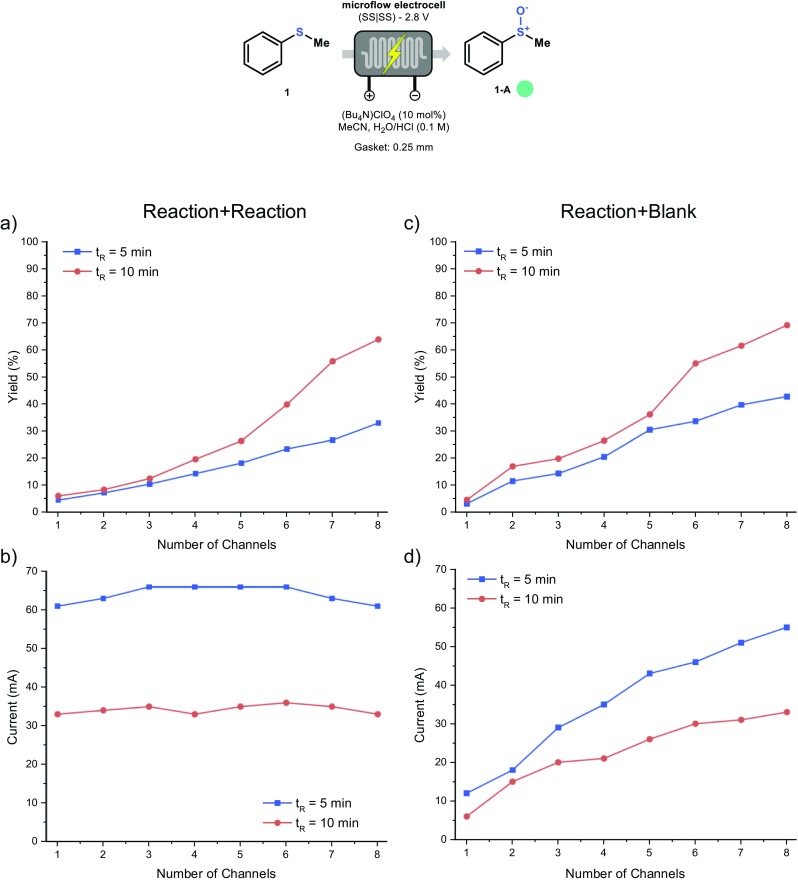
Experiment with gradually increasing channel numbers in series. The non-reactive channels are either filled with reaction mixture (**a**–**b**) or acetonitrile (**c**–**d**). Both the current (**b**–**d**) and the conversion (**a**–**c**) was recorded for every experiment. Residence time refers to the 8-channel configuration (last data point in the graph). Yield determined by GC-MS with biphenyl as internal standard

Next, we tested the electrochemical reactor in the serial mode by placing increasing numbers of channels in series. Using this strategy, the reactor volume can be systematically increased by 88 μL/channel, when a gasket of *d*_*G*_ = 0.25 mm is used. The non-participating channels were filled with either reaction mixture or acetonitrile. As can be seen from Fig. 9a and b, the sulfoxide yield systematically increases with an increasing number of channels, while the current remained stable during the entire experiment when the non-participating channels were filled with reaction mixture (Fig. 9b). In contrast, when the non-participating channels were filled with solvent (Fig. 9c and d), an increase in current was detected when the number of channels filled with the reaction mixture was increased (Fig. 9d). The yield, however, increased similarly in both scenarios.

**Scheme 1:**
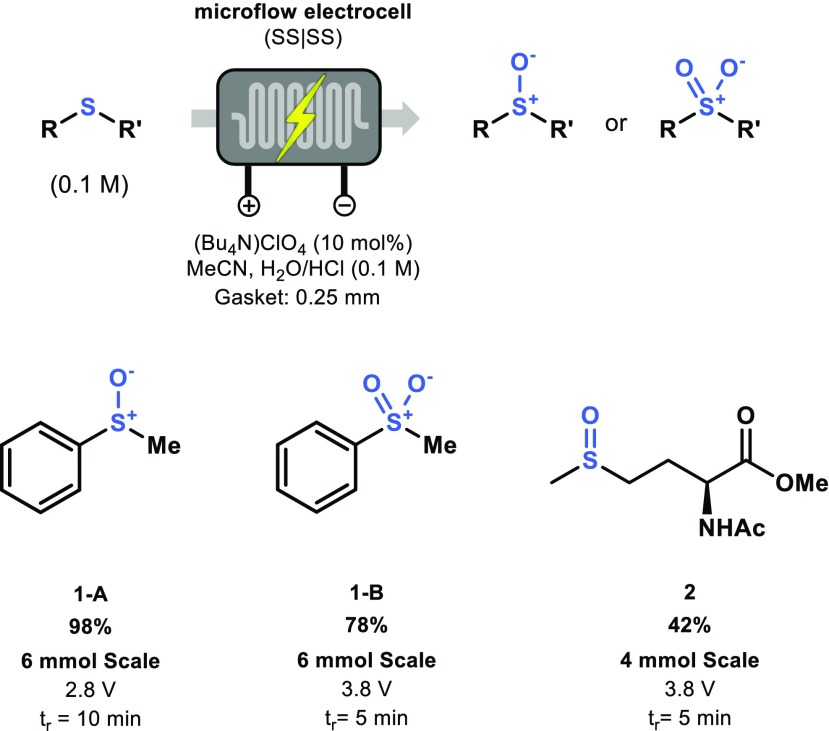
Preparative scale of **1-A** and **1-B** and **3** via an electrochemical anodic oxidation of thioethers

Having established insight in the governing parameters, we set out to probe the synthetic utility of the electrochemical flow cell. The preparative synthesis of compounds **1-A** and **1-B** was carried out with the 8-channel configuration and the two products could be isolated with respective yields of 98% and 78% at a 6 mmol scale (Scheme 1a). Furthermore, the bioactive molecule methionine sulfoxide (**2**) could be isolated in a 42% yield using a 5 min residence time (Scheme 1b).

**Scheme 2:**
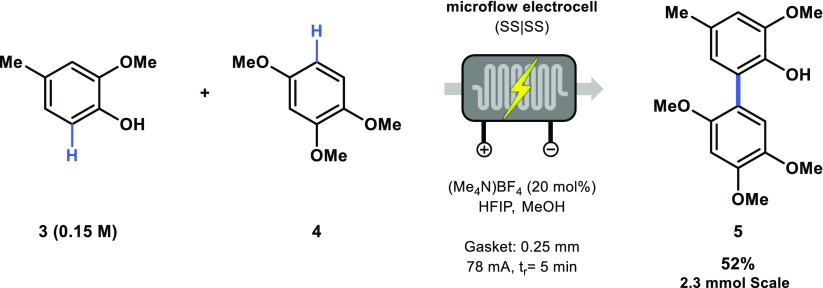
Preparative scale of **5** via an electrochemical arene-phenol cross coupling method

In order to further demonstrate the robustness of our electrochemical microreactor, we focused our attention on the electrochemical arene-phenol cross-coupling transformation as developed by Waldvogel et al. (Scheme 2) [28, 29]. Employing the 8-channel configuration, the corresponding biaryl **5** was obtained in a 52% isolated yield on a 2.3 mmol scale. While slightly lower yields were obtained in comparison with the original report, we were able to use a cheap and easily accessible stainless steel anode instead of the more expensive boron doped diamond anode [28, 29, 67].

## Conclusion

Herein, we have described and validated a novel, undivided-cell electrochemical flow reactor. The reactor is modular and can be fabricated with straightforward machining techniques. A unique feature of this reactor is the flexible reactor volume which can be used in a serial (volume ranging from 88 μL/channel up to 704 μL) or parallel mode (i.e. numbering-up). The electrochemical flow reactor was subsequently assessed in two synthetic transformations, which confirms its versatility and scale-up potential. Application of this reactor in other electrochemical transformations is currently pursued in our lab and will be reported in due course.

## Electronic supplementary material

ESM 1 (PDF 563 kb)

ESM 2 (PDF 2518 kb)

ESM 3 (PDF 664 kb)
