# Supplementary material for: Design and application of a modular and scalable electrochemical flow microreactor
Source: J Flow Chem. 2018 Nov 22;8(3):157–65. doi: 10.1007/s41981-018-0024-3 (PMC6404740; doi:10.1007/s41981-018-0024-3)
Supplement: Supplementary file 1 — (PDF 563 kb) [file 41981_2018_24_MOESM1_ESM.pdf]

Supporting Information (SI)

Design and Application of a Modular and Scalable Electrochemical Flow Microreactor

Gabriele Laudadio, Wouter de Smet, Lisa Struik, Yiran Cao, Timothy Noël*

Department of Chemical Engineering and Chemistry, Micro Flow Chemistry & Synthetic Methodology, Eindhoven University of Technology, De Rondom 70 (Helix, STO 1.37), 5612 AP Eindhoven, The Netherlands.

Website: www.NoelResearchGroup.com

E-mail: t.noel@tue.nl

Table of Contents

1. General information 3

2. Reactor characterization 4

Reactor Volume 4

3. Chemical validation of the reactor 5

3.1. Voltammogram 5

3.2. Residence time experiment 6

3.3. Single channel experiment 7

3.4. Multiple channels experiment 8

4. General procedure for continuous-flow reactions 9

4.1. Sulfide oxidation 9

4.2. Arene-phenol cross coupling 10

4.3. Faraday efficiency 11

5. Characterization data 14

6. Voltammograms 15

7. References 16

8. NMR Spectra 17

1. General information

All reagents and solvents were used as received without further purification, unless stated otherwise. Reagents and solvents were bought from Sigma Aldrich and TCI and if applicable, kept under argon atmosphere. Technical solvents were bought from VWR International and Biosolve, and are used as received. All capillary tubing and microfluidic fittings were purchased from IDEX Health & Science. Disposable syringes were from BD Discardit II® or NORM-JECT®, purchased from VWR Scientific. Syringe pumps were purchased from Chemix Inc. model Fusion 200 Touch. Product isolation was performed manually, using silica (60, F254, Merck™) or automatically by a Biotage® Isolera Four, with Biotage® SNAP KP-Sil 10 or 25 g flash chromatography cartridges. The temperature of the system was detected with a Voltcraft K204 thermomether, equipped with a 0.25 mm thick thermocouple. TLC analysis was performed using Silica on aluminum foils TLC plates (F254, Supelco Sigma-Aldrich™) with visualization under ultraviolet light (254 nm and 365 nm) or appropriate TLC staining. ^{1}H (400MHz) and ^{13}C (100MHz) spectra were recorded on ambient temperature using a Bruker-Avance 400 or Mercury 400. ^{1}H NMR spectra are reported in parts per million (ppm) downfield relative to $CDCl_3$ (7.26 ppm) and all ^{13}C NMR spectra are reported in ppm relative to $CDCl_3$ (77.2 ppm) unless stated otherwise. NMR spectra uses the following abbreviations to describe the multiplicity: s = singlet, d = doublet, t = triplet, q = quartet, p = pentet, h = hextet, hept = heptet, m = multiplet, dd = double doublet, td = triple doublet. NMR data was processed using the MestReNova 9.0.1 software package. Known products were characterized by comparing to the corresponding ^{1}H NMR and ^{13}C NMR from literature. GC analyses were performed on a GC-MS combination (Shimadzu GC-2010 Plus coupled to a Mass Spectrometer; Shimadzu GCMS-QP 2010 Ultra) with an auto sampler unit (AOC-20i, Shimadzu). Melting points were determined with a Buchi B-540 capillary melting point apparatus in open capillaries and are uncorrected. The names of all products were generated using the PerkinElmer ChemBioDraw Ultra v.12.0.2 software package.

For all electrochemical reactions, the newly designed flow cell was used, together with a Velleman LABPS3005D power supply that is connected to the flow cell. The cell consists of a working electrode and a counter electrode, both made of stainless steel, with a PTFE (Polytetrafluoroethylene) gasket containing micro-channels in between. Depending on whether a 0.25 mm or 0.5 mm thick gasket is used, the active reactor volume is either 700µL or 1300µL. This results in an undivided electrochemical cell. In the cell, direct contact between the electrode surface and the reaction mixture is established. The reaction mixture is pumped through the system via syringe pump, and is collected in a glass vial. Both electrodes can be set to be the anode or the cathode at any time.

All the technical data of the electrochemical setup are reported in the second file of the Supporting Information.

2. Reactor characterization

Reactor Volume

Table S1: Average volume measurement for the 8-channel gaskets tested

	Gasket 0.25 mm	Gasket 0.50 mm
**Channel Volume (8x)**	88 µL	164 µL
**Tube Volume (7x)**	106 µL	106 µL
**Standard Deviation**	7.5 µL	16.7 µL
**Active Reactor Volume**	**704 µL**	**1310 µL**
**Active Reactor Surface**	**26 cm^2**	**26 cm^2**
**Total Tube Volume**	744 µL	744 µL
**Total Reactor Volume**	1446 µL	2054 µL
**Standard Deviation %**	6.9%	9.0%

A syringe was filled with acetonitrile and connected to the electrochemical setup. After totally filling the cell with fluid, a small bubble was created in the syringe connector. By setting the syringe pump to a set flowrate (which was varied from 0.1 mL/min to 1 mL/min), this bubble could be tracked while flowing through the tubes. The time was noted when the bubble front entered and exited the cell through each fitting, resulting in an amount of time spend inside each channel and outside of the reactor. By multiplying these times with the flowrate, the volumes between the inlets and outlets of each channel can be determined. As the inlet holes of the electrode and the tubes within the fittings have a set volume of 23.2 µL, this was subtracted from the channel volume and added to the tube volume. The total active volume was determined by adding all channel volumes together. The total reactor volume is the sum of all channels and tubes.

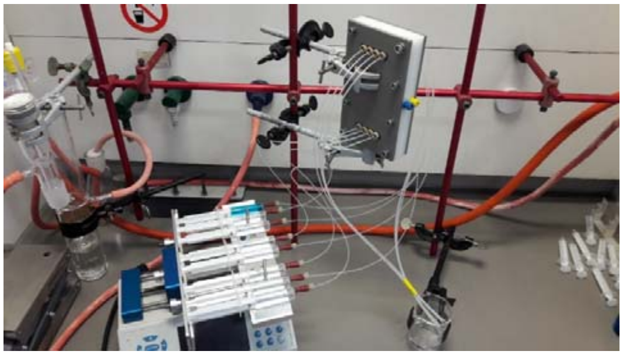

Figure S 1 Reactor volume experiment

3. Chemical validation of the reactor

3.1. Voltammogram

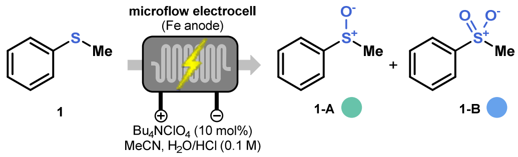

Thioanisole (**1**) was dissolved in the corresponding amount of stock solution (3:1 v/v acetonitrile/0.1 M HCl (aq) and 10 mol% tetrabutylammonium perchlorate (Bu_4NClO_4) to yield a 0.1 M solution of thioether in stock solution. The solution was flown through the electrochemical setup with a fixed flowrate of 0.15 mL/min to give a residence time of 5 minutes in the active part of the reactor, using the 0.25mm thick gasket. During the experiment, the potential was varied between 2.0 V – 4.0 V. Samples were taken at the corresponding potential after the total residence time had elapsed (10min at 0.15 mL/min). For each fraction, the current was determined. For quantitative analysis, every sample was analysed without further purification using GC-MS or GC-FID with an internal standard (biphenyl). During the experiment, the temperature of the reaction mixture was recorded with a thermocouple placed at the end of the reactor via T-mixer. No consistent change of the temperature was detected (20.4 °C).[1]

Analogue experiment was carried out to record the Voltammogram in the Syrris Asia Flux device, as reported in the previous work.[2]

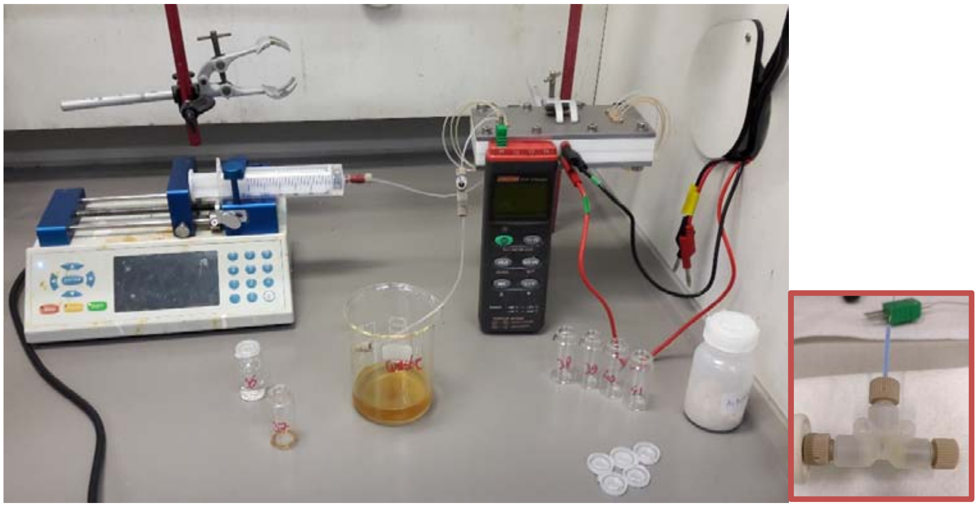

Figure S 2 Voltammogram experiment. In detail: T-mixer equipped with thermocouple.

3.2. *Residence time experiment*

Thioanisole (**1**) was dissolved in the corresponding amount of stock solution (3:1 v/v acetonitrile/0.1 M HCl (aq) and 10 mol% tetrabutylammonium perchlorate (Bu_4NClO_4) to yield a 0.1 M solution of thioether in stock solution. The solution was flown through the electrochemical setup at a flowrate ranging from 0.05 mL/min up to 0.35 mL/min to give a residence time of 2 to 14 minutes in the active part of the reactor. During the experiment, the potential was set at the optimal potential found in the screening experiment (3.1 V) and an intermediate potential (2.8 V). Samples were taken for each flowrate after 2 residence times had elapsed. For each fraction, the current was determined. For quantitative analysis, every sample was analysed without further purification using GC-MS or GC-FID with an internal standard (biphenyl).

3.3. *Single channel experiment*

Thioanisole (**1**) was dissolved in the corresponding amount of stock solution (3:1 v/v acetonitrile/0.1 M HCl (aq) and 10 mol% tetrabutylammonium perchlorate (Bu_4NClO_4) to yield a 0.1 M solution of thioether in stock solution. The reactor was rebuild in order to have for every channel an inlet and an outlet, obtaining an 8-channel pattern. The solution was pumped through the electrochemical setup at a flowrate of 0.05 mL/min, leading to a residence time of 1.75 min for every channel. The potential was set at 2.8 V and samples were taken for each channel after 2 residence times had elapsed. For each fraction, the current was determined. For quantitative analysis, every sample was analysed without further purification using GC-MS or GC-FID with an internal standard (biphenyl).

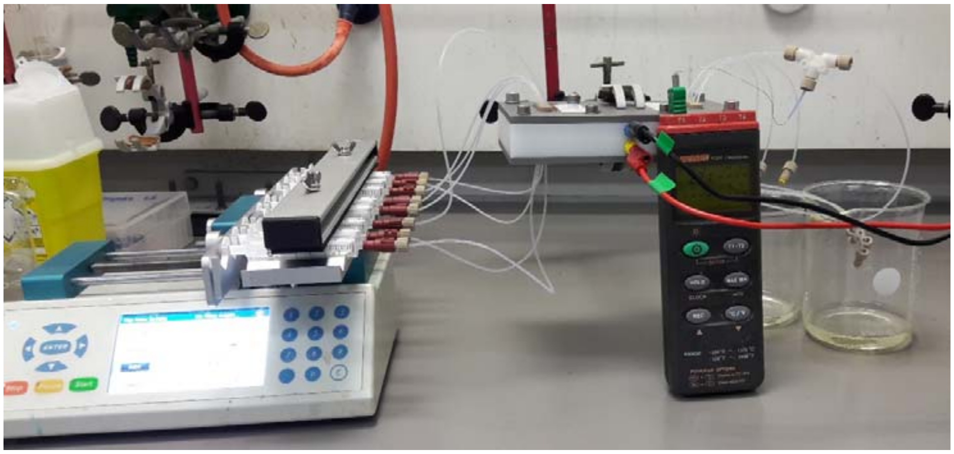

Figure S 3 Single channel experiment

3.4. Multiple channels experiment

Thioanisole (**1**) was dissolved in the corresponding amount of stock solution (3:1 v/v acetonitrile/0.1 M HCl (aq) and 10 mol% tetrabutylammonium perchlorate (Bu_4NClO_4) to yield a 0.1 M solution of thioether in stock solution. The solution was pumped through the electrochemical setup with a flowrate of 0.15 mL/min and 0.075 mL/min to give a residence time of 5 minutes and 10 minutes in the active part of the reactor respectively. During the experiment, the potential was set at 2.8 V. Two syringes were connected to the reactor and the amount of channels per syringe was varied by changing the tube loops around. For the full reaction test both syringes would be filled with reaction mixture. For the reaction + blank test, one of the syringes would be filled with acetonitrile. Samples were taken for each amount of channels (ranging for just one channel to all eight channel) after 2 residence times had elapsed. For quantitative analysis, every sample was analysed without further purification using GC-MS or GC-FID with an internal standard (biphenyl).

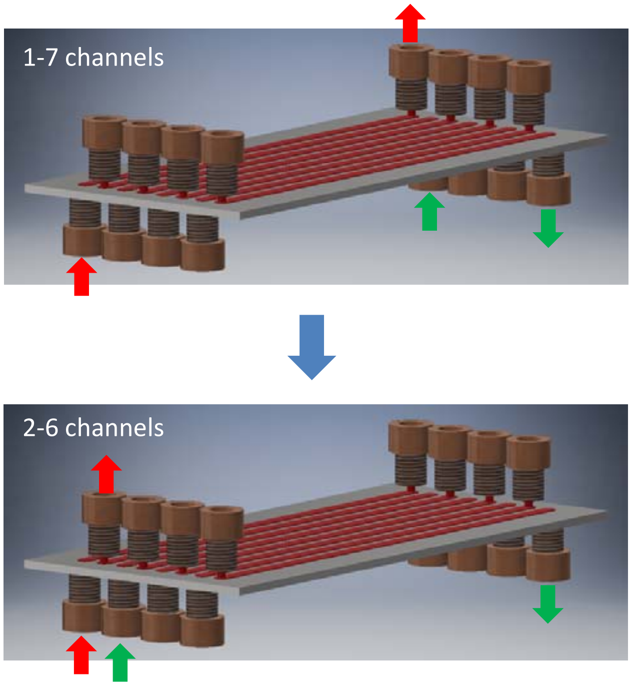

Figure S 4 Representation of the multiple channels experiment. Red and green arrows represent the two different streams.

4. General procedure for continuous-flow reactions

4.1. Sulfide oxidation

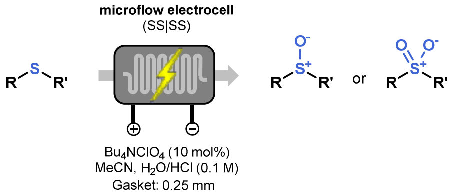

4.1.1. Optimal potential determination

The sulfide was dissolved in the corresponding amount of stock solution (3:1 v/v acetonitrile/0.1M HCl (aq), 10 mol% Bu_4NClO_4) to yield a total solution of 0.1 M towards starting material. The solution was pumped through the electrocell with a fixed flowrate of 0.15 mL/min to give the residence time in the active part of 5 minutes. The potential was varied from 2.2 V to 4.2 V. After 2 residence times had elapsed, the corresponding current was noted and a sample was collected. Every sample was analysed without further purification using GC-MS or GC-FID.

4.1.2. Preparative scale

The sulfide was dissolved in the corresponding amount of stock solution (3:1 v/v acetonitrile/0.1M HCl (aq), 10 mol% Bu_4NClO_4) to yield a total solution of 0.1 M towards starting material. The solution was pumped through the electrochemical setup at the potential determined in the screening. The reaction mixture was collected in an Erlenmeyer flask. The crude was diluted with saturated sodium bicarbonate solution (15 mL) and transferred to a separatory funnel. The mixture was extracted with ethyl acetate (3 x 20 mL), dried over magnesium sulfate and concentrated in vacuo. The product was then purified using column chromatography (cyclohexane/ethyl acetate) and analysed by TLC, GC-MS, ^{1}H-NMR and ^{13}C-NMR.

4.2. Arene-phenol cross coupling

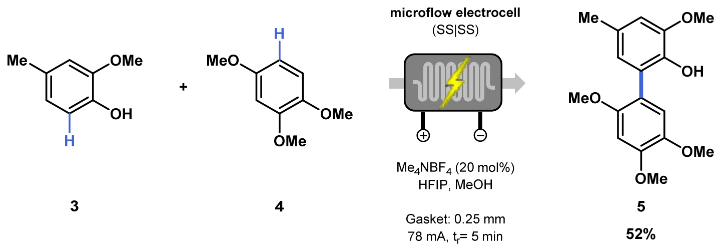

4.2.1. Optimal current determination

A solution of 1,2,4-trimethoxybenzene (0.015 mol), 2-methoxy-4-methylphenol (0.005 mol) and Me_4NBF_4 (0.0007 mol) in 1,1,1,3,3,3-hexafluoropropan-2-ol (27 mL) and methanol (6 mL) was pumped through the electrocell with a fixed flowrate of 0.15 mL/min to give a residence time of 5 minutes. The current was varied from 10 mA to 100 mA. After 2 residence times had elapsed (10 minutes at 0.15 mL/min), the corresponding potential was noted and a sample was collected. Every sample was analysed without further purification using GC-MS or GC-FID.

4.2.2. Preparative scale

A solution of 1,2,4-trimethoxybenzene (0.015 mol), 2-methoxy-4-methylphenol (0.005 mol) and tetramethylammonium tetrafluoroborate (0.007 mol) in 1,1,1,3,3,3-hexafluoropropan-2-ol (27 mL) and methanol (6 mL) was pumped through the electrocell with a fixed flowrate of 0.15 mL/min to give a residence time of 5 minutes. The reaction mixture was collected in an Erlenmeyer flask. The crude was diluted with saturated sodium bicarbonate solution (15 mL) and transferred to a separatory funnel. The mixture was extracted with ethyl acetate (3 x 20 mL), dried over magnesium sulfate and concentrated in vacuo. The product was then purified using column chromatography (cyclohexane/ethyl acetate 4:1) and analysed by TLC, GC-MS, ^{1}H-NMR and ^{13}C-NMR.

4.3. *Faraday efficiency*

4.3.1. Sulfide oxidation

Assuming a complete anodic oxidation for the sulfoxide (2 e^- per mole of substrate) and sulfone (4 e^- per mole of substrate)

Average current for the oxidation to sulfoxide

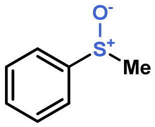

1-A

$I = 46\ mA$

$r_t = 600\ s$

$Yield = 98\%$

$Q = I * r_t = 27.6\ C$

Experimental electron amount

$$n_{e_{expe}} = \frac{Q}{F} = \frac{27.6\ C}{96485\ C * mol^{-1}} = 0.286\ mmol$$

Theoretical electron amount

$V_{reactor} = 0.702\ mL$

$C_{substrate} = 0.1\ M$ $\qquad$ $n_{substrate} = 0.07\ mmol$

$n_{e_{theo}} = 0.14\ mmol$ $\qquad$ (assuming 2 electrons needed)

Faraday Efficiency

$$F_{efficiency}\% = \frac{n_{e_{theo}}}{n_{e_{expe}}} * 100 = \frac{0.14\ mmol}{0.286\ mmol} * 100 = 49\ \%$$

$$F_{yield}\% = F_{efficiency}\% * yield = 48\ \%$$

Average current for the oxidation to sulfone

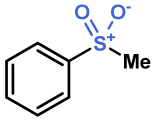

1-B

$I = 146\ mA$

$r_t = 300\ s$

$Yield = 78\%$

$Q = I * r_t = 43.8\ C$

Experimental electron amount

$$n_{e_{expe}} = \frac{Q}{F} = \frac{43.8\ C}{96485\ C * mol^{-1}} = 0.454\ mmol$$

Theoretical electron amount

$V_{reactor} = 0.702\ mL$

$C_{substrate} = 0.1\ M$ $n_{substrate} = 0.07\ mmol$

$n_{e_{theo}} = 0.28\ mmol$ (assuming 4 electrons needed)

Faraday Efficiency

$$F_{efficiency}\% = \frac{n_{e_{theo}}}{n_{e_{expe}}} * 100 = \frac{0.28\ mmol}{0.454\ mmol} * 100 = 62\ \%$$

$$F_{yield}\% = F_{efficiency}\% * yield = 48\ \%$$

4.3.2. Biphenyl coupling oxidation

Assuming a complete anodic oxidation for the sulfoxide (2 e^- per mole of substrate) and sulfone (4 e^- per mole of substrate)

Average current for the oxidation to sulfoxide

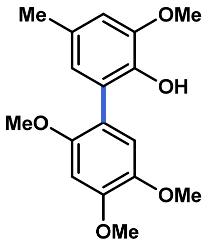

5

$$I = 78\ mA$$
$$r_t = 300\ s$$
$$Yield = 52\%$$
$$Q = I * r_t = 23.4\ C$$

Experimental electron amount

$$n_{e_{expe}} = \frac{Q}{F} = \frac{23.4\ C}{96485\ C * mol^{-1}} = 0.242\ mmol$$

Theoretical electron amount

$V_{reactor} = 0.702\ mL$

$C_{substrate} = 0.15\ M$ $n_{substrate} = 0.105\ mmol$

$n_{e_{theo}} = 0.21\ mmol$ (assuming 2 electrons needed)[3]

Faraday Efficiency

$$F_{efficiency}\% = \frac{n_{e_{theo}}}{n_{e_{expe}}} * 100 = \frac{0.21\ mmol}{0.242\ mmol} * 100 = 87\%$$

$$F_{yield}\% = F_{efficiency}\% * yield = 45\ \%$$

5. Characterization data

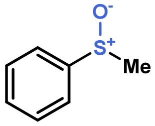

1-A

(Methylsulfinyl)benzene (1-A).[2] Following the general procedure (4.1.1-4.1.2, 6 mmol scale), (methylsulfinyl)benzene was obtained from thioanisole at 2.8 V pumping the reaction mixture through the reactor twice at 5 minutes residence time per run, yielding 830 mg of product (5.9 mmol, 98%) as a yellow solid.

^{1}H-NMR (399 MHz, Chloroform-*d*) δ 7.68 – 7.62 (m, 2H), 7.57 – 7.45 (m, 3H), 2.73 (s, 3H) ppm. ^{13}C{^{1}H} NMR (100 MHz, Chloroform-*d*) δ 146.1, 131.4, 129.7, 123.8, 44.3 ppm.

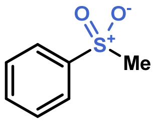

1-B

(Methylsulfonyl)benzene (1-B).[2] Following the general procedure (4.1.1-4.1.2, 6 mmol scale), (methylsulfonyl)benzene was obtained from thioanisole at 3.8 V yielding 640 mg of product (4.1 mmol, 78%) as a white solid.

^{1}H NMR (399 MHz, Chloroform-*d*) δ 7.98 – 7.90 (m, 2H), 7.70 – 7.62 (m, 1H), 7.61 – 7.52 (m, 2H), 3.05 (s, 3H) ppm. ^{13}C{^{1}H} NMR (100 MHz, Chloroform-*d*) δ 140.9, 134.0, 129. 7, 127.7, 44.8 ppm.

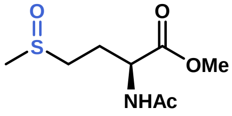

3

Methyl (2*S*)-2-acetamido-4-(methylsulfinyl)butanoate (3).[2] Following the general procedure (4.1.1-4.1.2, 4 mmol scale), methyl (2*S*)-2-acetamido-4-(methylsulfinyl)butanoate was obtained from methyl acetyl-L-methioninate at 3.8 V pumping the reaction mixture through the reactor twice at 5 minutes residence time per run, yielding 372 mg of product (1.68 mmol, 42%) as an off-white solid.

1H NMR (399 MHz, Chloroform-d) δ 6.97 (dd, J = 31.7, 7.4 Hz, 1H), 4.65 (tt, J = 8.0, 4.5 Hz, 1H), 3.73 (s, 3H), 2.86 – 2.60 (m, 2H), 2.56 (d, J = 1.8 Hz, 3H), 2.38 – 2.25 (m, 2H), 2.00 (d, J = 2.5 Hz, 3H). ^{13}C{^{1}H} NMR (100 MHz, Chloroform-*d*) δ 171.9, 171.8, 170.6, 170.6, 77.5, 77.2, 76.8, 52.6, 52.6, 51.2, 51.0, 50.3, 50.1, 38.4, 38.4, 25.7, 25.2, 22.9, 22.9.

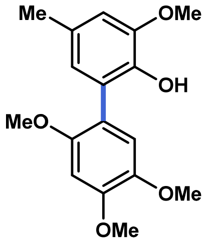

5

2',3',4',5'-tetramethoxy-5-methyl-[1,1'-biphenyl]-2-ol (5).[3] Following the protocol (4.2.1-4.2.2), 2',3',4',5'-tetramethoxy-5-methyl-[1,1'-biphenyl]-2-ol was obtained at 78 mA, yielding 702 mg of product (2.3 mmol, 52%) as an white-off solid.

^{1}H NMR (399 MHz, Chloroform-*d*) δ 6.85 (s, 1H), 6.73 – 6.67 (m, 2H), 6.65 (s, 1H), 3.92 (d, *J* = 11.1 Hz, 6H), 3.86 (s, 3H), 3.80 (s, 3H), 2.33 (s, 3H) ppm. ^{13}C{^{1}H} NMR (100 MHz, Chloroform-*d*) δ 150.5, 149.4, 147.6, 143.8, 141.0, 129.4, 125.3, 123.5, 118.7, 115.2, 111.4, 98.6, 57.4, 56.6, 56.3, 56.2, 21.3 ppm.

6. Voltammograms

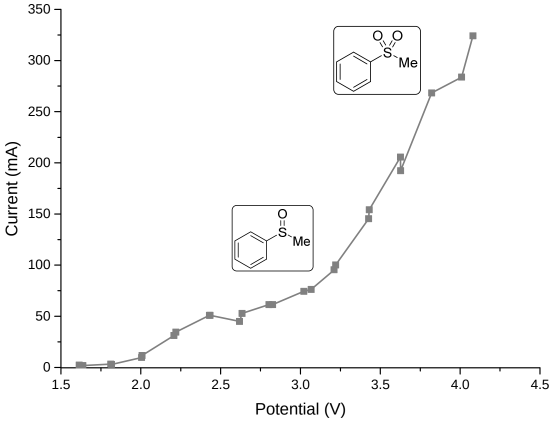

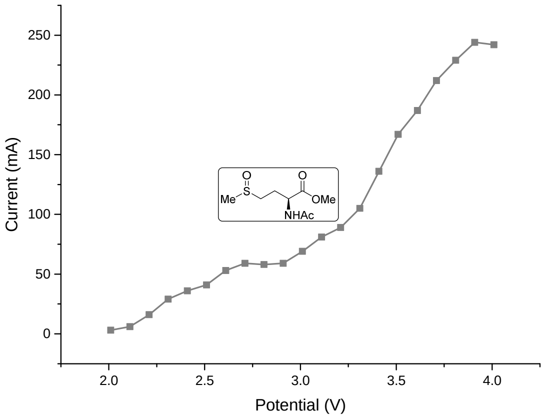

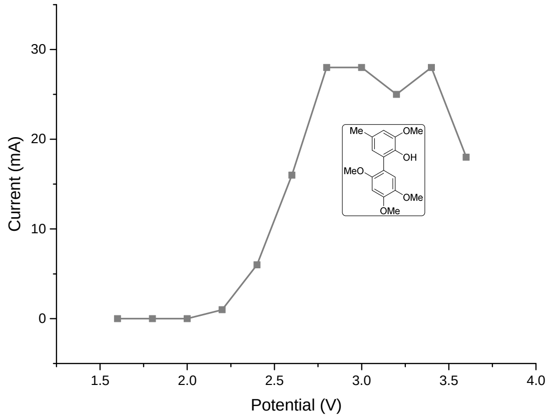

7. References

(1) Laudadio, G.; Gemoets, H. P. L.; Hessel, V.; Noël, T. *J. Org. Chem.* **2017**, *82* (22), 11735–11741.

(2) Laudadio, G.; Straathof, N. J. W.; Lanting, M. D.; Knoops, B.; Hessel, V.; Noël, T. *Green Chem.* **2017**, *19* (17), 4061–4066.

(3) Kirste, A., Elsler, B., Schnakenburg, G. & Waldvogel, S. R. *J. Am. Chem. Soc.* **2012** 3571–3576.

8. NMR Spectra

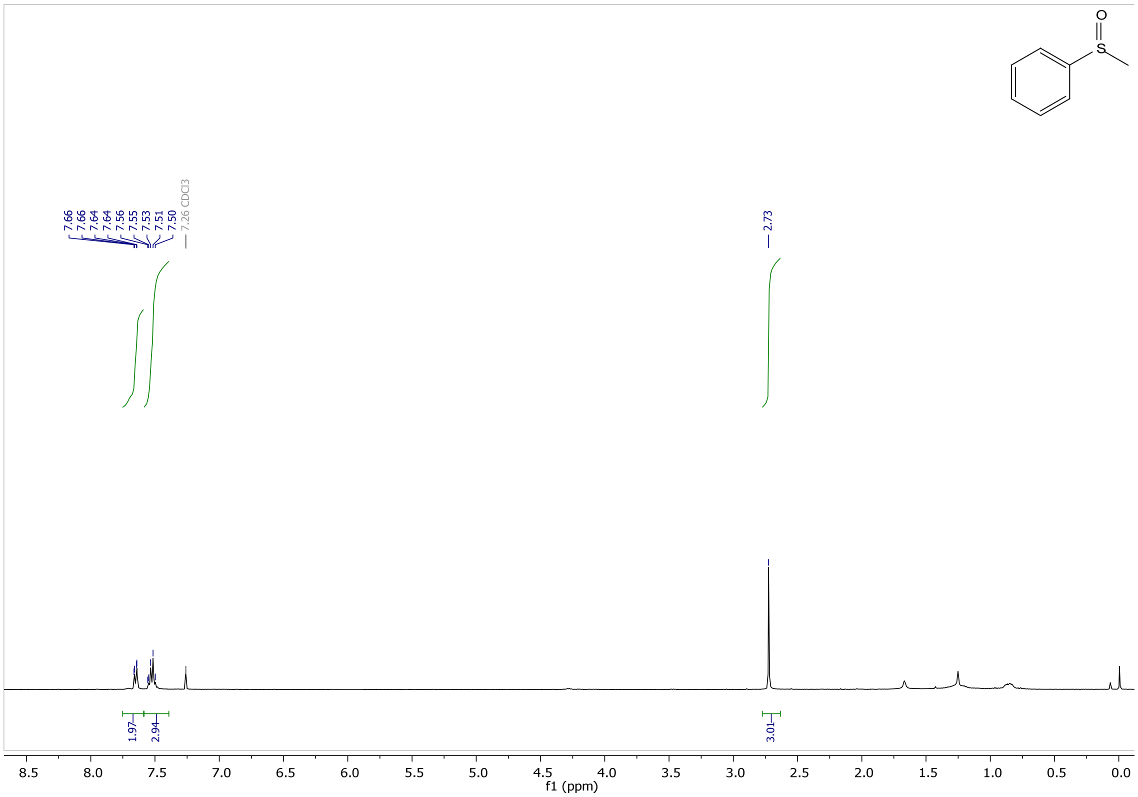

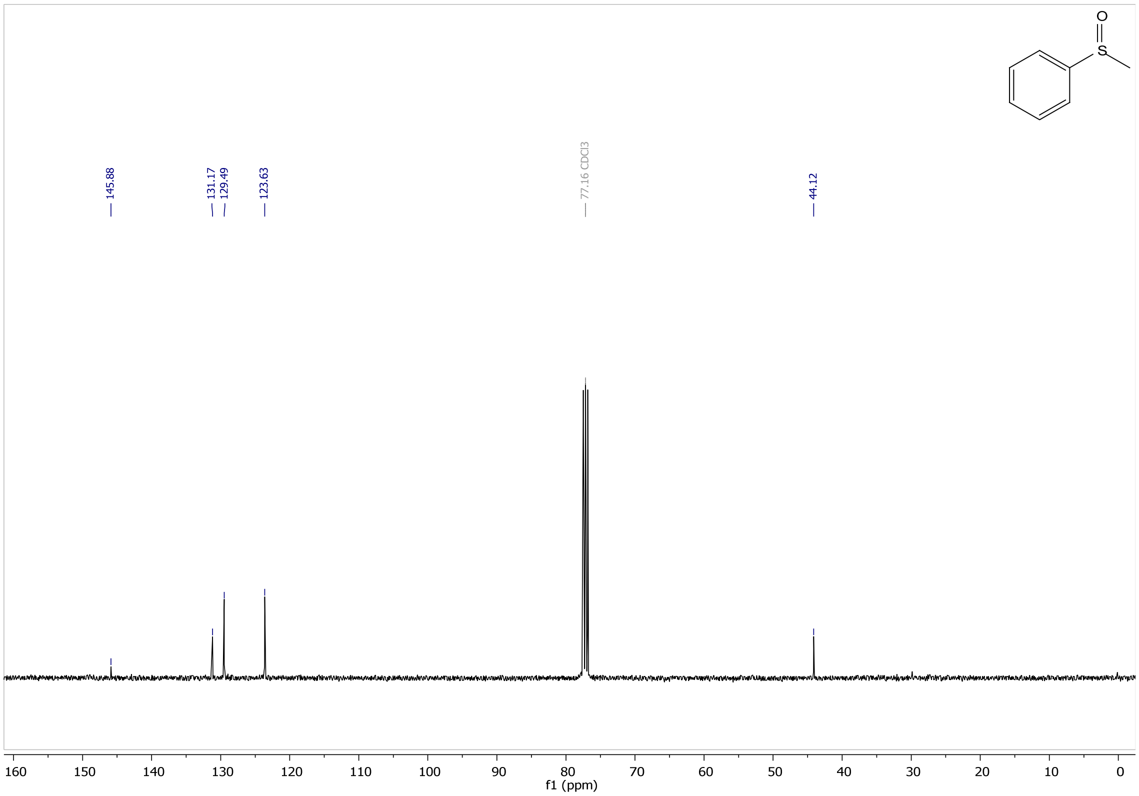

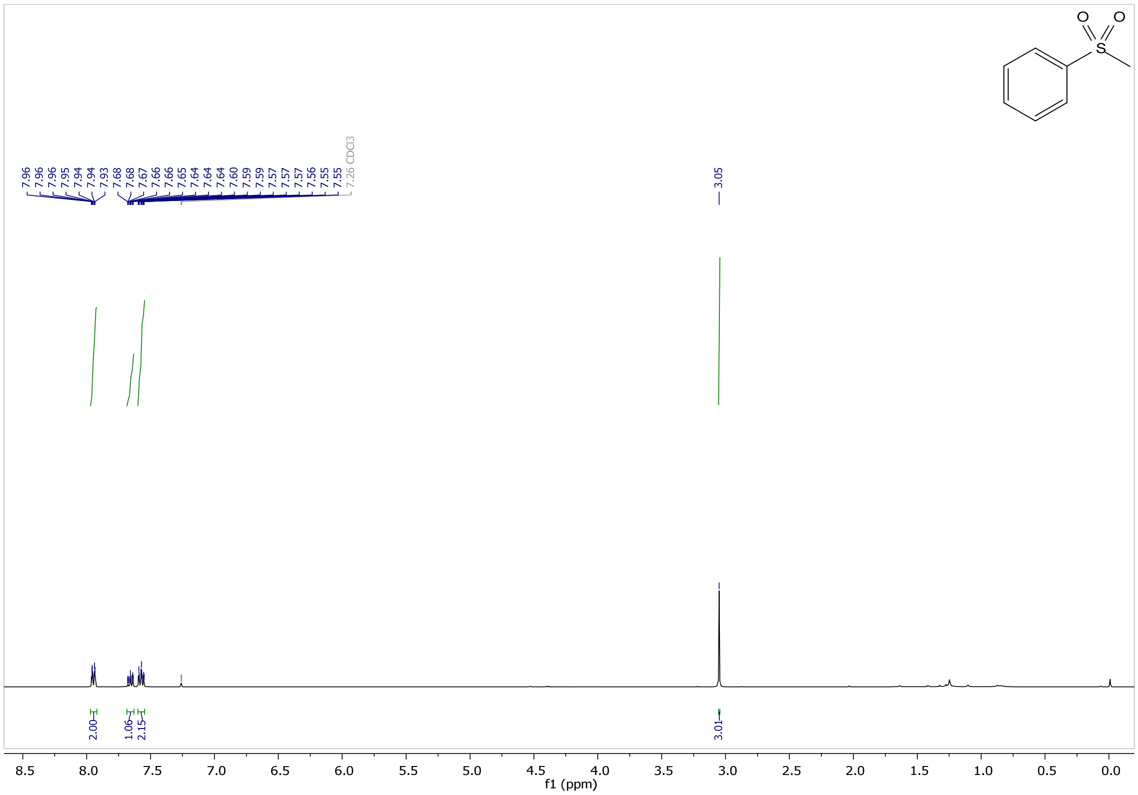
7.96
7.96
7.95
7.94
7.94
7.93
7.68
7.68
7.67
7.66
7.66
7.65
7.64
7.64
7.64
7.60
7.59
7.59
7.57
7.57
7.57
7.56
7.55
7.55
7.26 CDCl3
3.05
2.00
1.06
2.15
3.01
8.5 8.0 7.5 7.0 6.5 6.0 5.5 5.0 4.5 4.0 3.5 3.0 2.5 2.0 1.5 1.0 0.5 0.0
f1 (ppm)

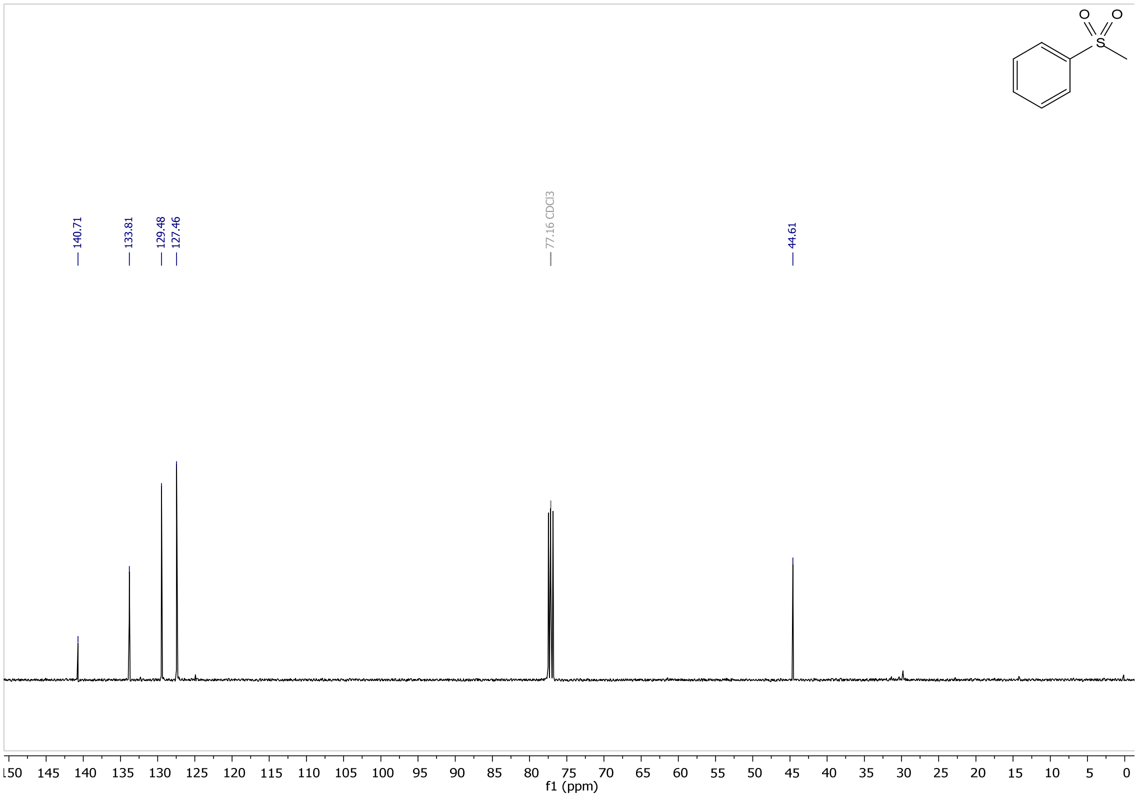
140.71
133.81
129.48
127.46
77.16 CDCl3
44.61
150 145 140 135 130 125 120 115 110 105 100 95 90 85 80 75 70 65 60 55 50 45 40 35 30 25 20 15 10 5 0
f1 (ppm)

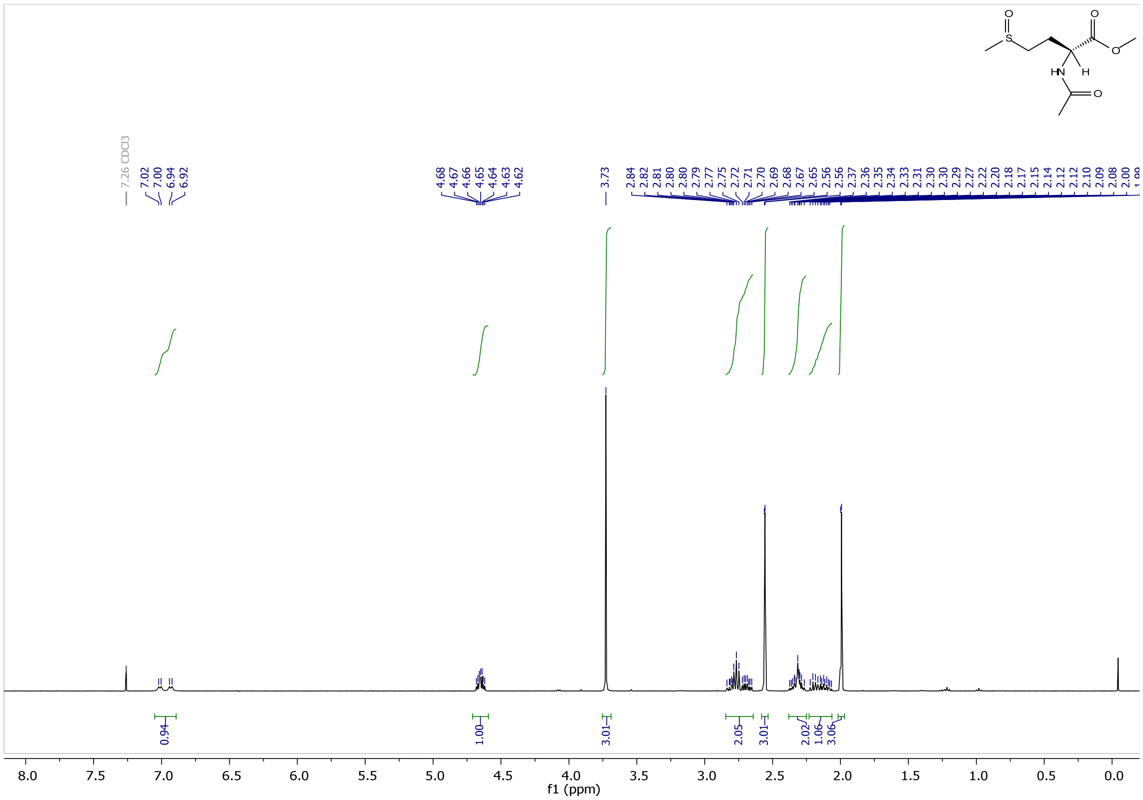
7.26 CDCl3
7.02
7.00
6.94
6.92
4.68
4.67
4.66
4.65
4.64
4.63
4.62
3.73
0.94
1.00
3.01
2.05
3.01
2.02
1.06
3.06
f1 (ppm)

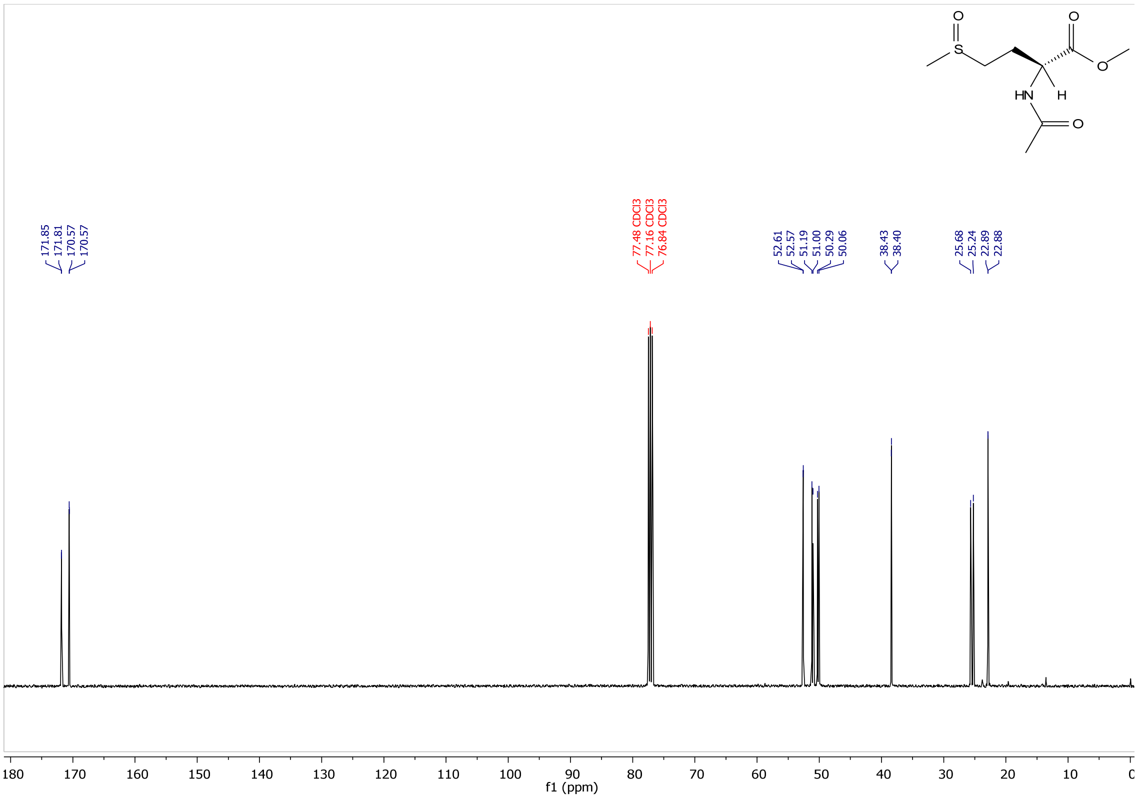
171.85
171.81
170.57
170.57
77.48 CDCl3
77.16 CDCl3
76.84 CDCl3
52.61
52.57
51.19
51.00
50.29
50.06
38.43
38.40
25.68
25.24
22.89
22.88
f1 (ppm)

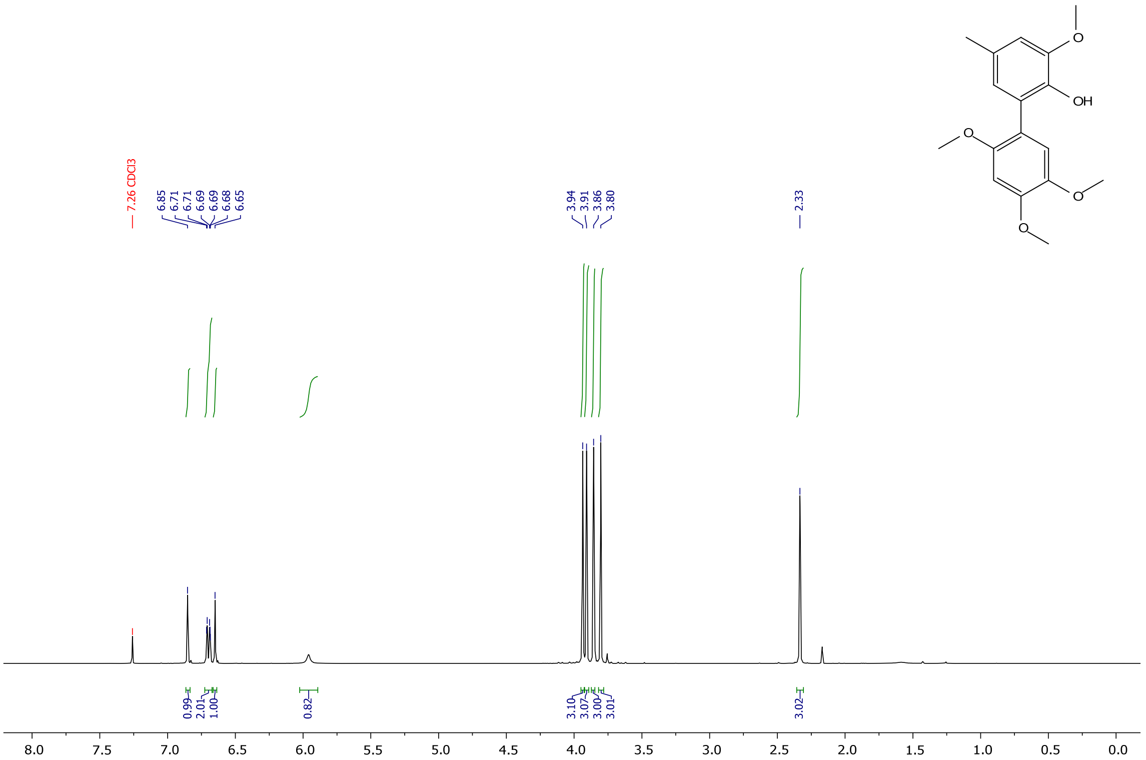
7.26 CDCl3
6.85
6.71
6.71
6.69
6.69
6.68
6.65
3.94
3.91
3.86
3.80
2.33
OH
0.99
2.01
1.00
0.82
3.10
3.07
3.00
3.01
3.02
8.0
7.5
7.0
6.5
6.0
5.5
5.0
4.5
4.0
3.5
3.0
2.5
2.0
1.5
1.0
0.5
0.0

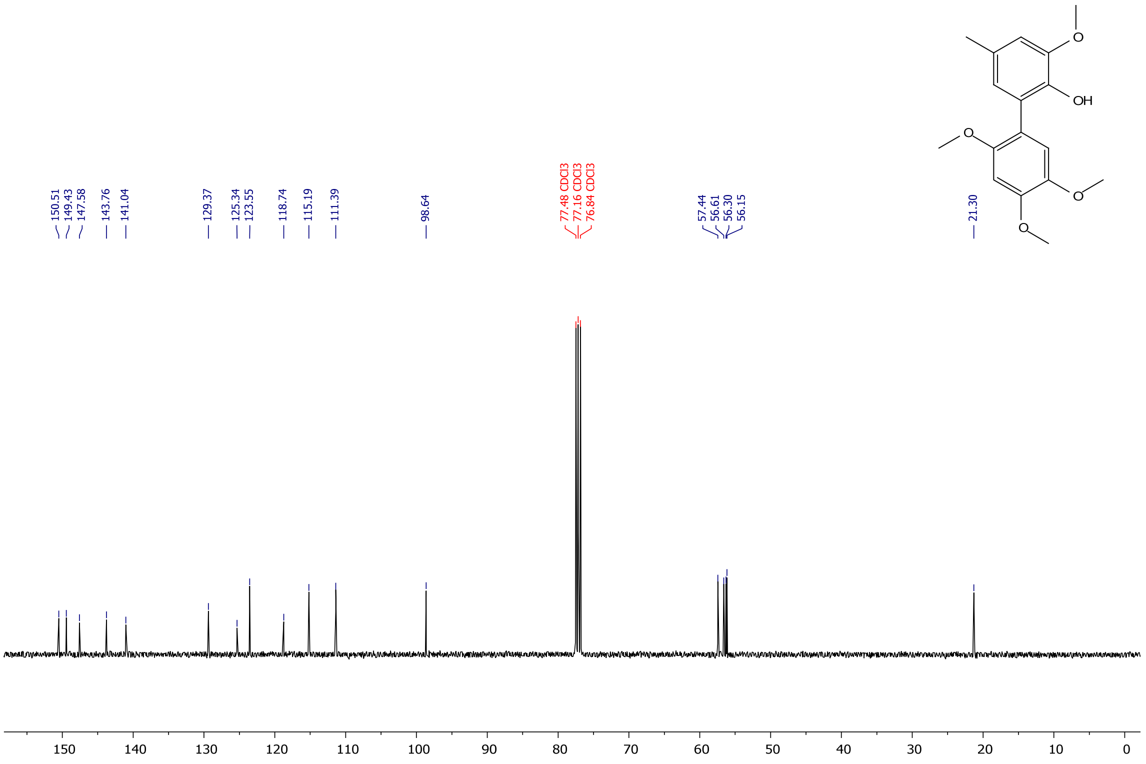
150.51
149.43
147.58
143.76
141.04
129.37
125.34
123.55
118.74
115.19
111.39
98.64
77.48 CDCl3
77.16 CDCl3
76.84 CDCl3
57.44
56.61
56.30
56.15
21.30
OH
150
140
130
120
110
100
90
80
70
60
50
40
30
20
10
0
