# Supplementary material for: Design and application of a modular and scalable electrochemical flow microreactor
Source: J Flow Chem. 2018 Nov 22;8(3):157–65. doi: 10.1007/s41981-018-0024-3 (PMC6404740; doi:10.1007/s41981-018-0024-3)
Supplement: Supplementary file 2 — (PDF 2518 kb) [file 41981_2018_24_MOESM2_ESM.pdf]

TITLE	
PART NO	Assembly
REVISION	
DESIGNER	s095679
ENGINEER	

NOTES

◀ ▶

THE INFORMATION AND/OR MATERIAL IN THIS DOCUMENT IS THE PROPERTY OF AND RESTRICTED INFORMATION AND/OR MATERIAL OF THE AUTHOR. THIS INFORMATION MAY NOT BE USED, REPRODUCED, PUBLISHED OR DISCLOSED TO OTHERS WITHOUT WRITTEN AUTHORIZATION. IT IS TO BE USED ONLY FOR MANUFACTURING ITEMS SPECIFIED WITHIN THE DOCUMENT.

v1.0
