# Supplementary material for: Design and application of a modular and scalable electrochemical flow microreactor
Source: J Flow Chem. 2018 Nov 22;8(3):157–65. doi: 10.1007/s41981-018-0024-3 (PMC6404740; doi:10.1007/s41981-018-0024-3)
Supplement: Supplementary file 3 — (PDF 664 kb) [file 41981_2018_24_MOESM3_ESM.pdf]

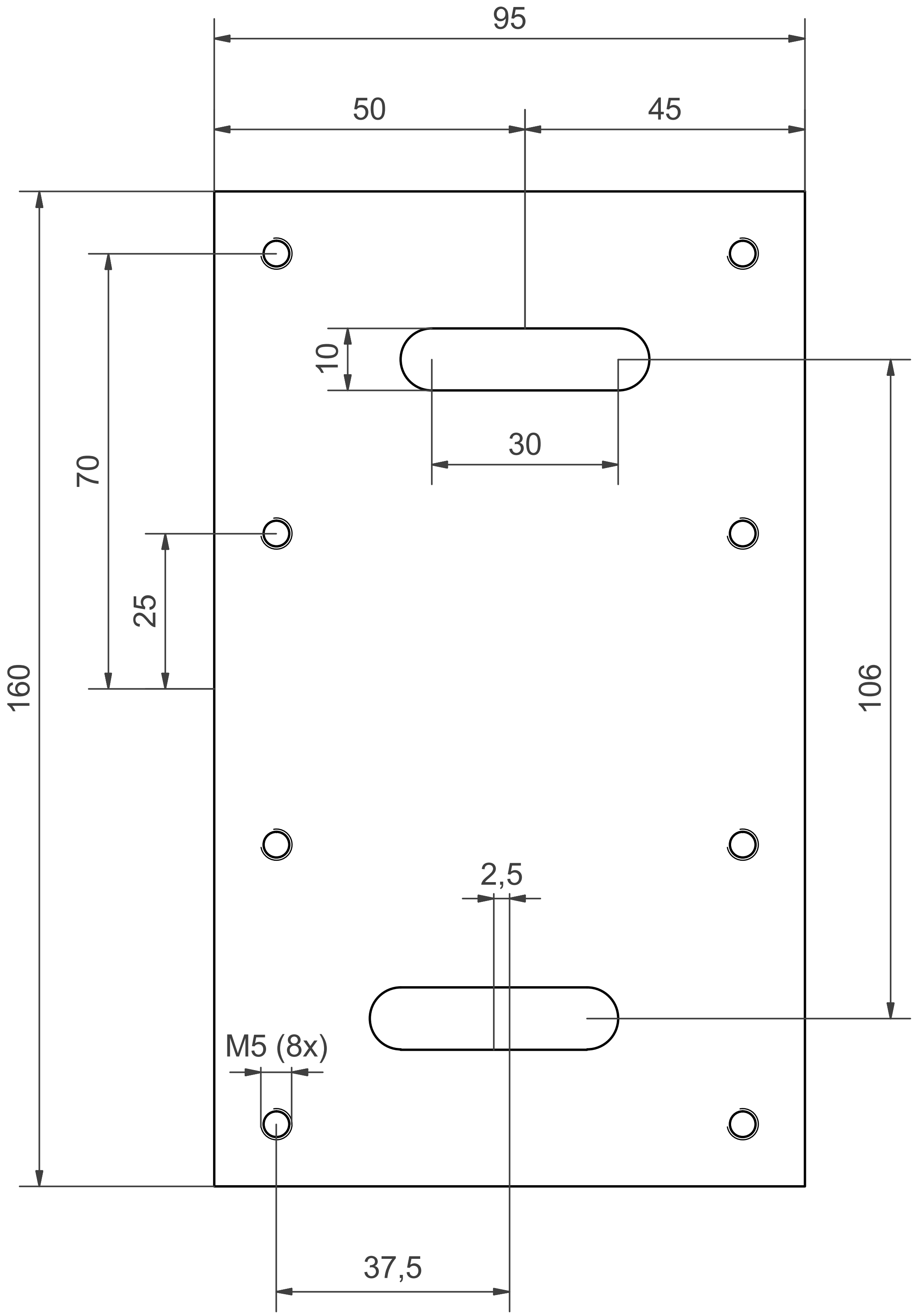

MAATTOLERANTIES, TENZIJ ANDERS VERMELD: ±0,2 mm
HOEKTOLERANTIES, TENZIJ ANDERS VERMELD: ±1°

PROJECTIE

BENAMING:

TU/e Technische Universiteit Eindhoven University of Technology

Equipment & Prototype Center

AANTAL:	MATERIAAL:		
SCHAAL:		PROJECTNUMMER :	A4
GET.:			
DATUM: **28-7-2017**		REVISIE:	

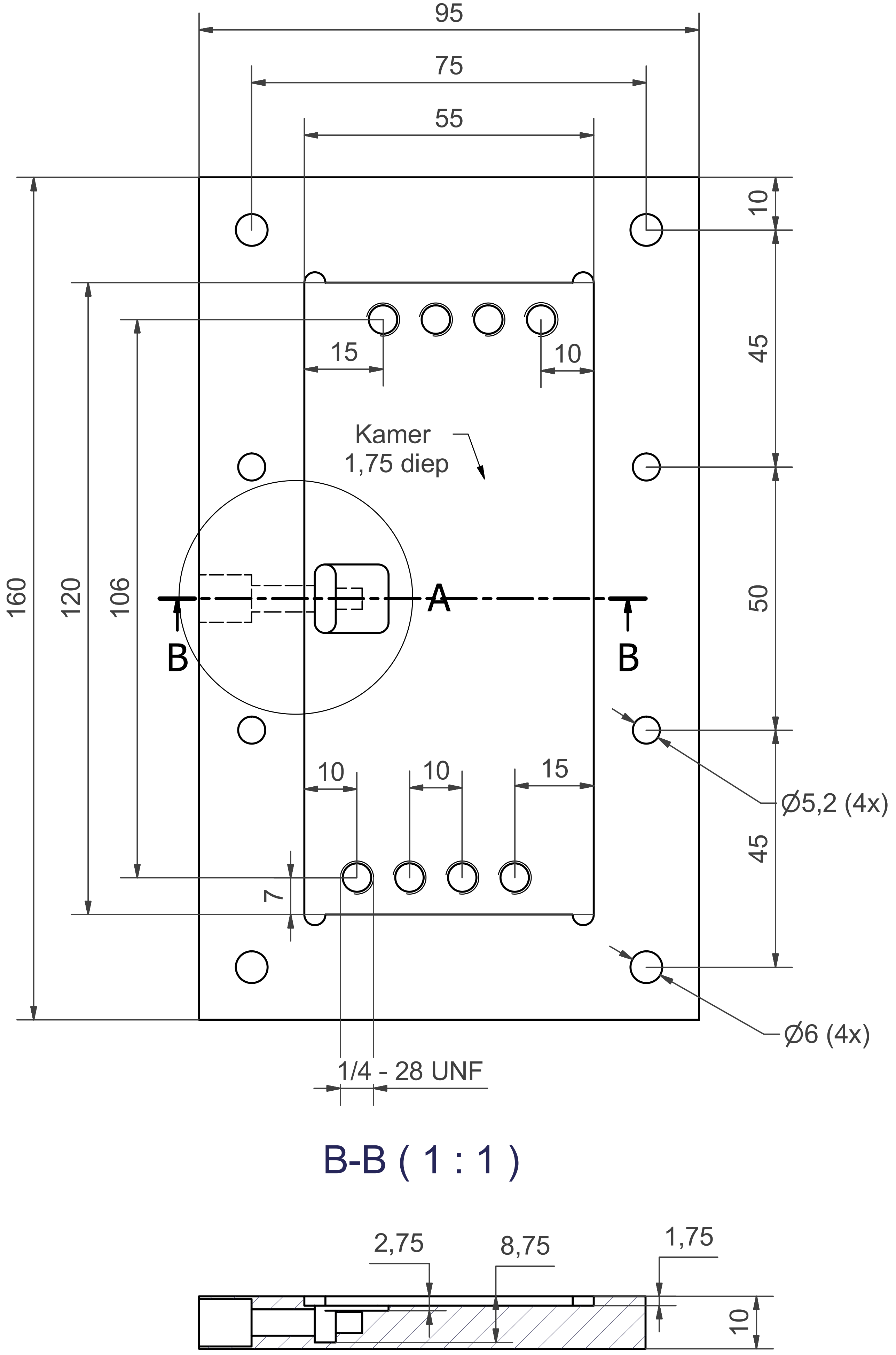

B-B (1 : 1)

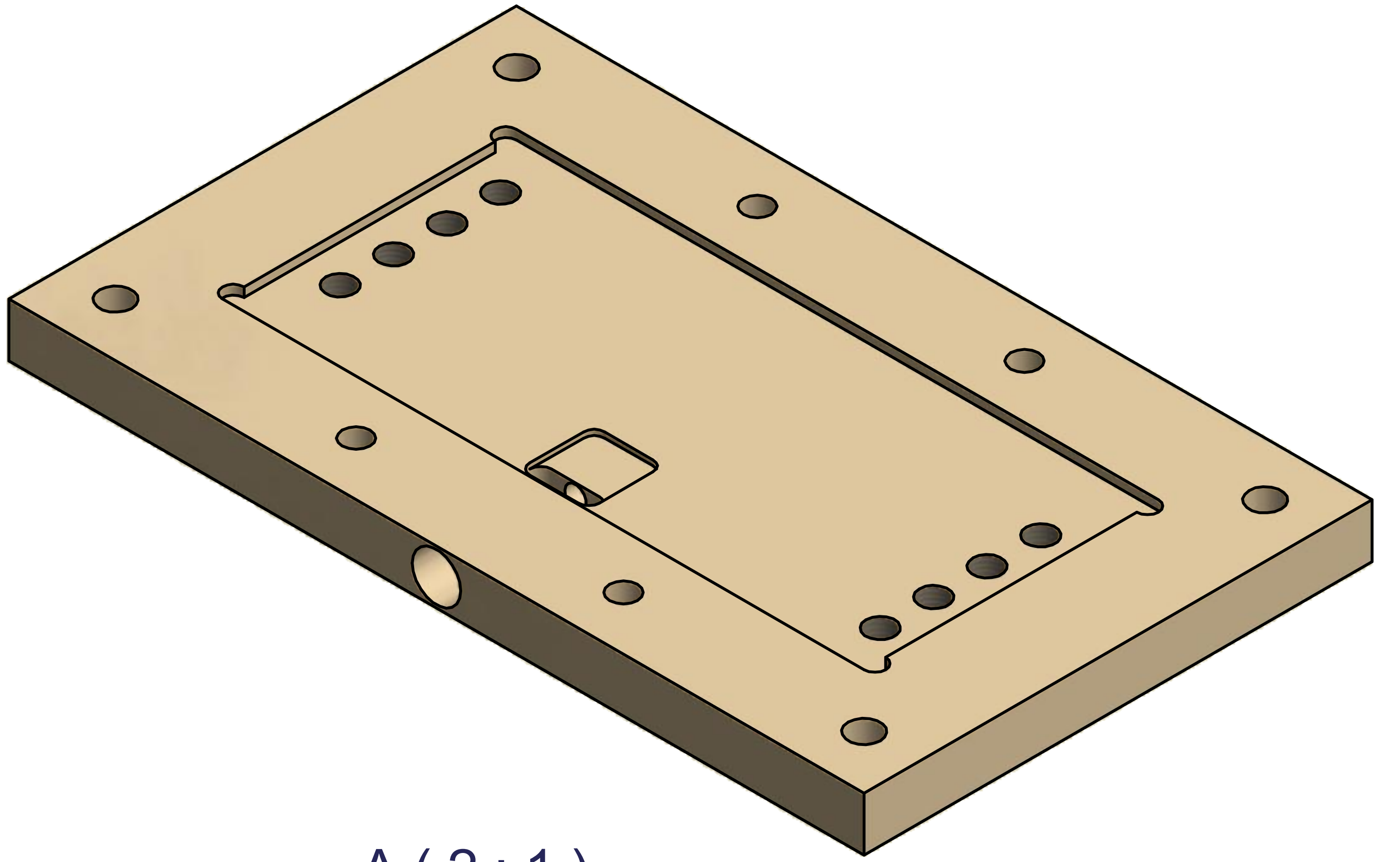

A (2 : 1)

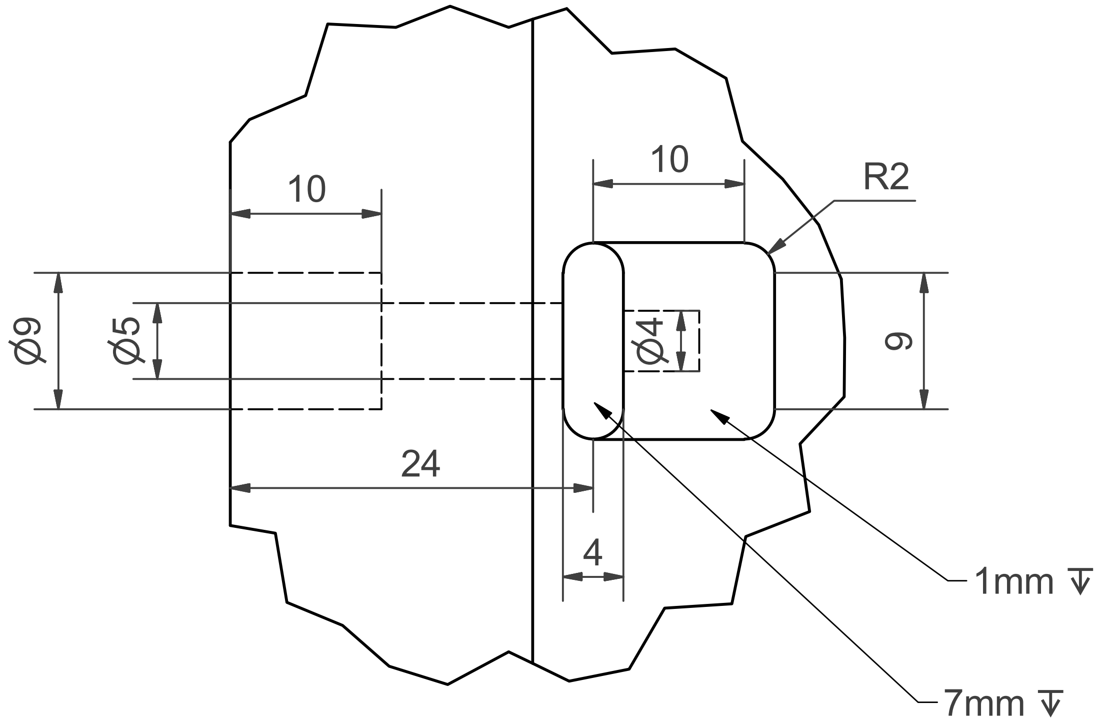

MAATTOLERANTIES, TENZIJ ANDERS VERMELD: ±0,2 mm<br>HOEKTOLERANTIES, TENZIJ ANDERS VERMELD: ±1°	PROJECTIE	BENAMING:<br>PTFE plate<br>**Electrochemische Microreactor**	
TU/e Technische Universiteit Eindhoven University of Technology<br>Equipment & Prototype Center	AANTAL: 2	MATERIAAL: PTFE	
	SCHAAL:	PROJECTNUMMER :	A3
	GET.: W Kuip		
	DATUM: 28-7-2017	REVISIE:	

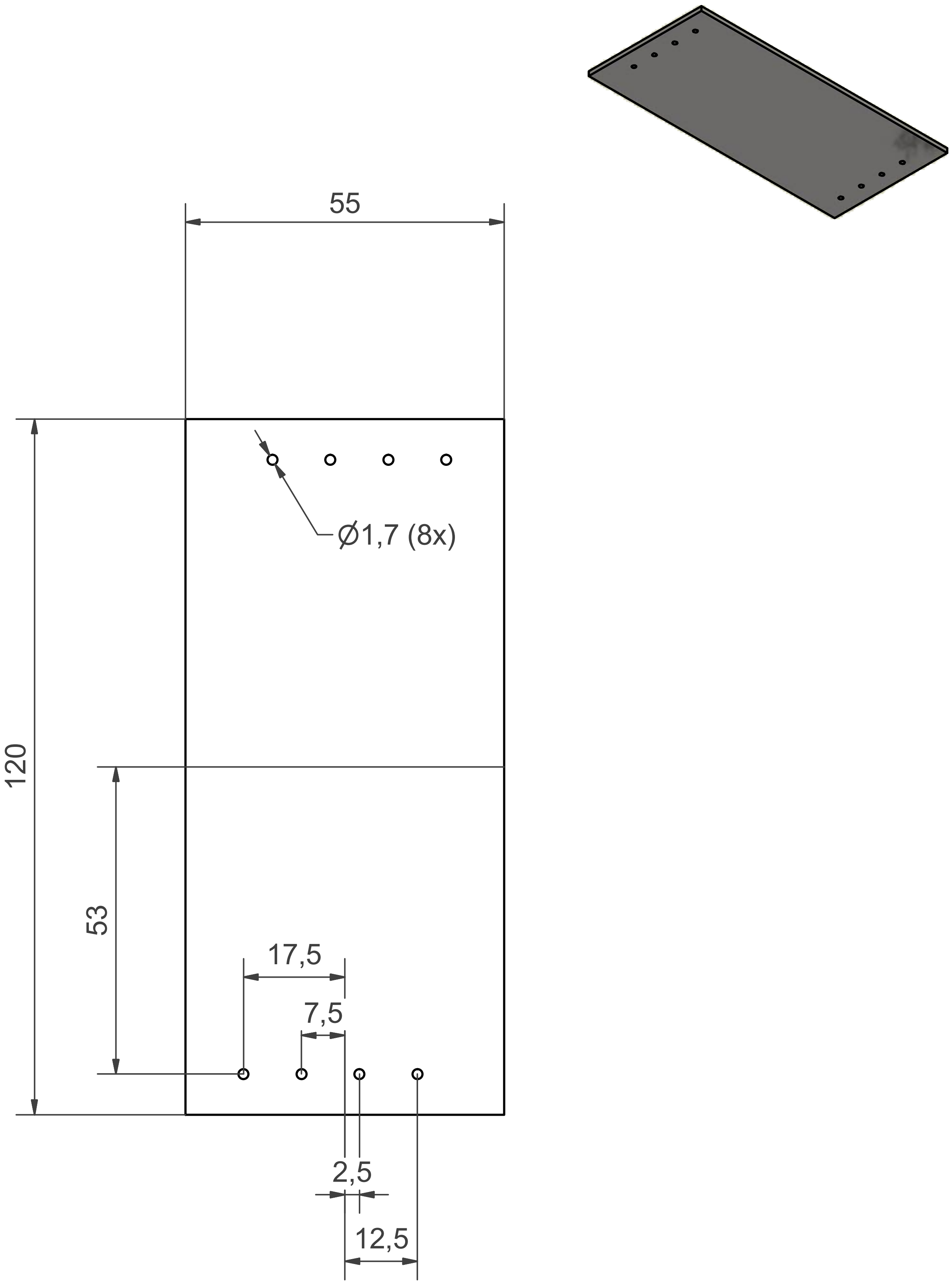

MAATTOLERANTIES, TENZIJ ANDERS VERMELD: ±0,2 mm
HOEKTOLERANTIES, TENZIJ ANDERS VERMELD: ±1°

PROJECTIE

BENAMING:

Technische Universiteit Eindhoven University of Technology

Equipment & Prototype Center

AANTAL:

MATERIAAL:

SCHAAL:

PROJECTNUMMER :

GET.:

DATUM: **28-7-2017**

REVISIE:

A4

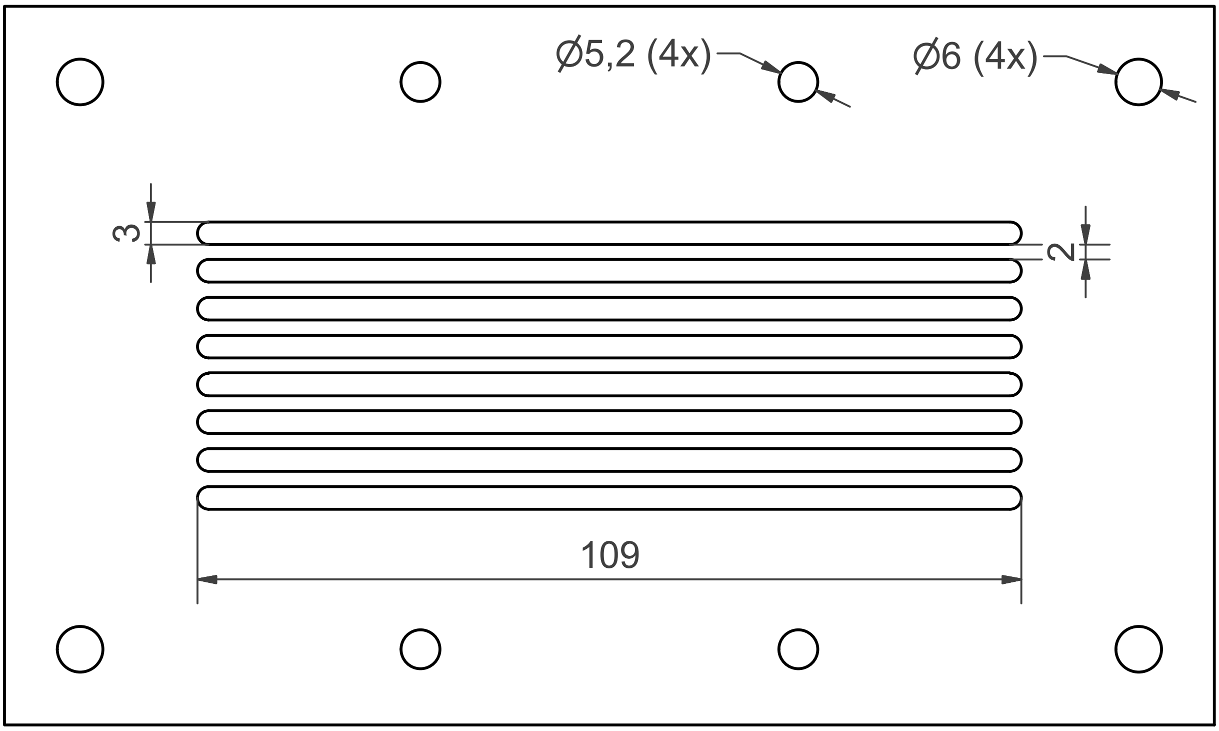

Teflon: ieder model 5 st 0,5 mm dik en 0,25 mm dik (totaal 20 st)

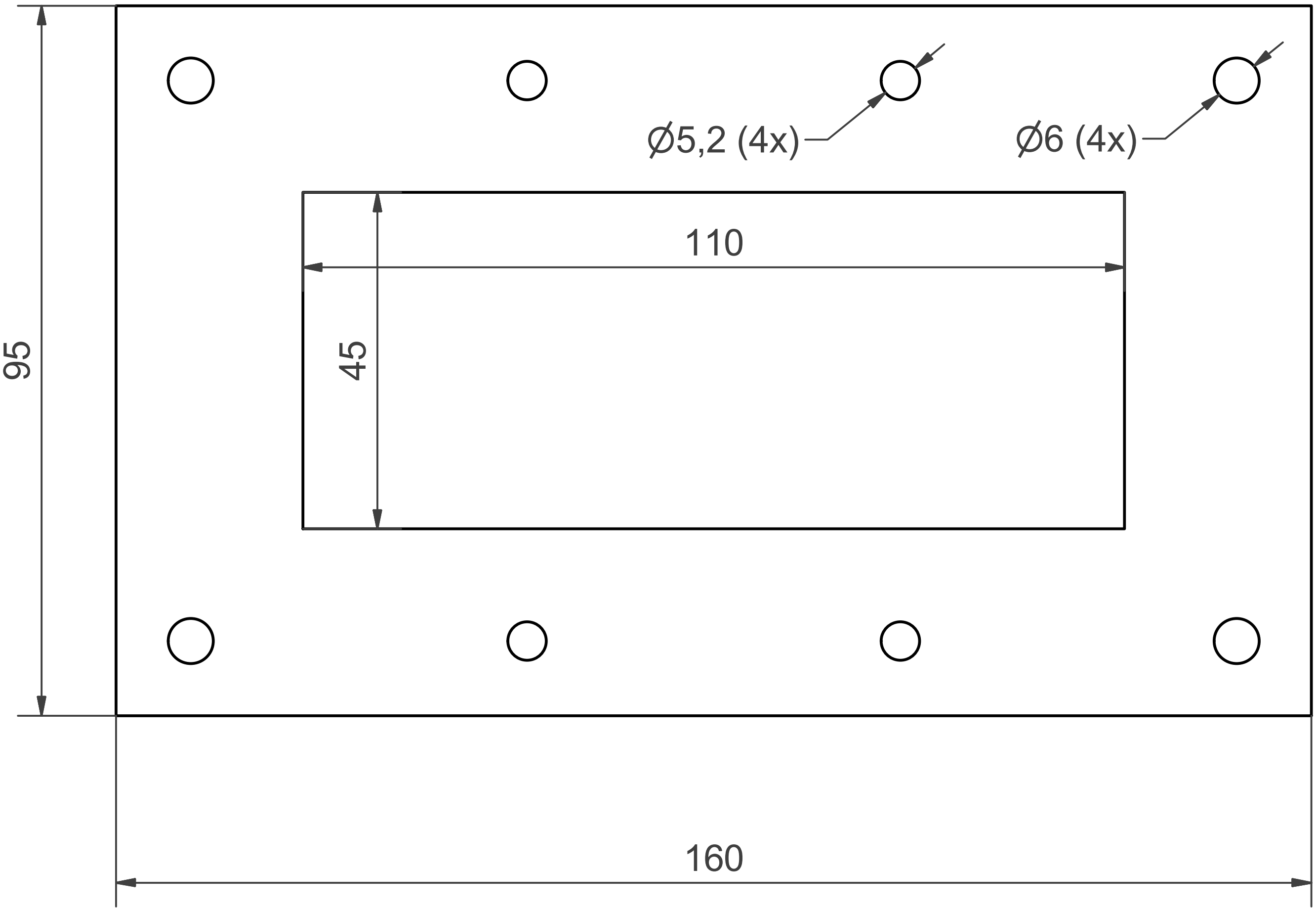

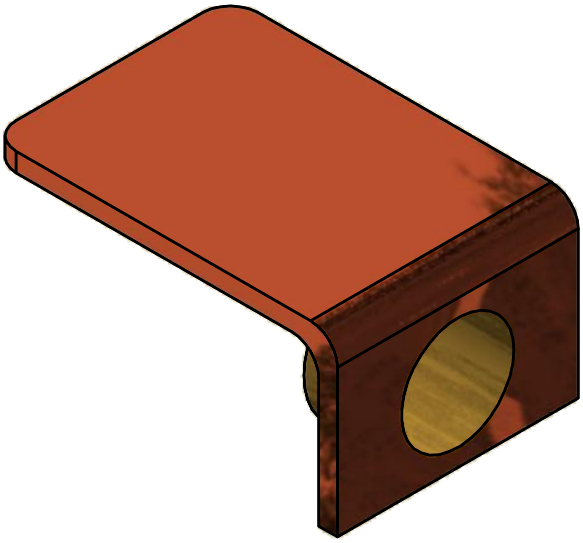

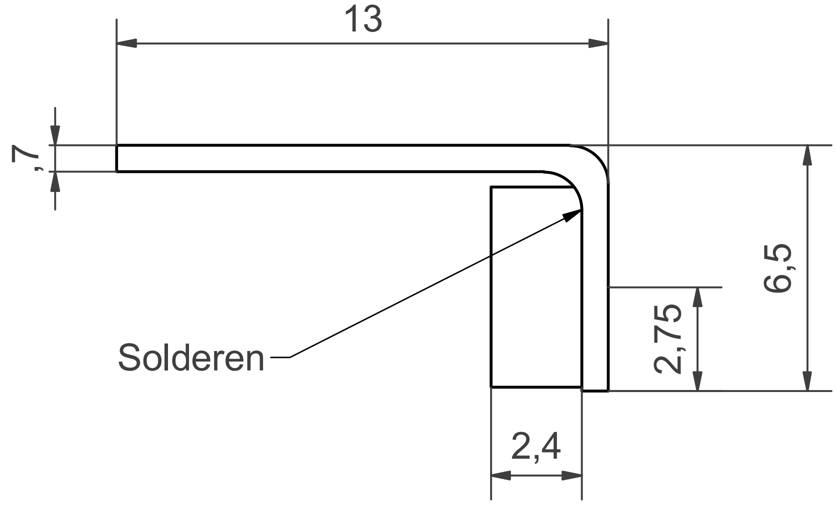

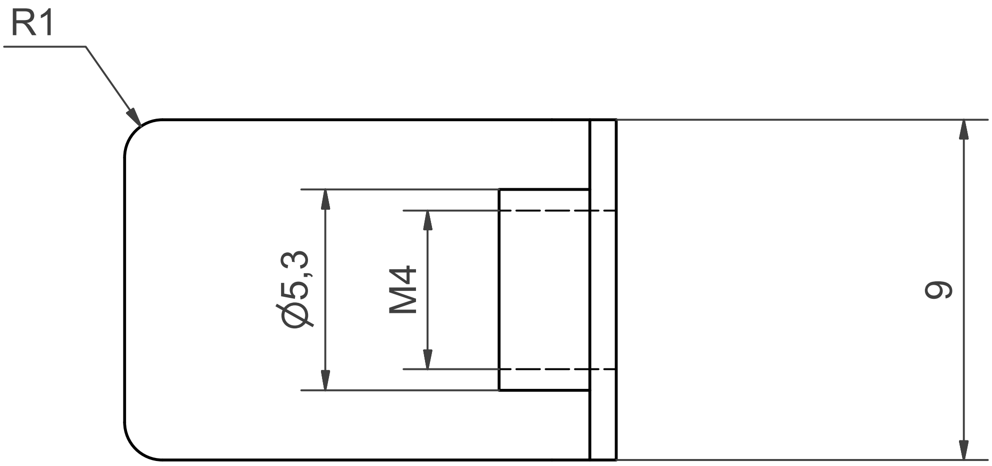

MAATTOLERANTIES, TENZIJ ANDERS VERMELD: ±0,2 mm<br>HOEKTOLERANTIES, TENZIJ ANDERS VERMELD: ±1°	PROJECTIE	BENAMING:<br>contact strip<br>**Elektrochemische Microreactor**			
TU/e Technische Universiteit Eindhoven University of Technology<br>**Equipment & Prototype Center**		AANTAL: **2**	MATERIAAL: **Fosforbrons**		
		SCHAAL:		PROJECTNUMMER :	A4
		GET.: **W. Kuip**			
		DATUM: **26-1-2018**		REVISIE:	
